# A Genetic RNAi Screen for IP_3_/Ca^2+^ Coupled GPCRs in *Drosophila* Identifies the PdfR as a Regulator of Insect Flight

**DOI:** 10.1371/journal.pgen.1003849

**Published:** 2013-10-03

**Authors:** Tarjani Agrawal, Sufia Sadaf, Gaiti Hasan

**Affiliations:** National Centre for Biological Sciences, Tata Institute of Fundamental Research, Bangalore, India; Washington University Medical School, United States of America

## Abstract

Insect flight is regulated by various sensory inputs and neuromodulatory circuits which function in synchrony to control and fine-tune the final behavioral outcome. The cellular and molecular bases of flight neuromodulatory circuits are not well defined. In *Drosophila melanogaster*, it is known that neuronal IP_3_ receptor mediated Ca^2+^ signaling and store-operated Ca^2+^ entry (SOCE) are required for air-puff stimulated adult flight. However, G-protein coupled receptors (GPCRs) that activate intracellular Ca^2+^ signaling in the context of flight are unknown in *Drosophila*. We performed a genetic RNAi screen to identify GPCRs that regulate flight by activating the IP_3_ receptor. Among the 108 GPCRs screened, we discovered 5 IP_3_/Ca^2+^ linked GPCRs that are necessary for maintenance of air-puff stimulated flight. Analysis of their temporal requirement established that while some GPCRs are required only during flight circuit development, others are required both in pupal development as well as during adult flight. Interestingly, our study identified the Pigment Dispersing Factor Receptor (PdfR) as a regulator of flight circuit development and as a modulator of acute flight. From the analysis of PdfR expressing neurons relevant for flight and its well-defined roles in other behavioral paradigms, we propose that PdfR signaling functions systemically to integrate multiple sensory inputs and modulate downstream motor behavior.

## Author Summary

A majority of behavioral patterns in flying insects depend upon their ability to modulate flight. In *Drosophila melanogaster*, mutations in the IP_3_ receptor gene lead to loss of voluntary flight in response to a natural stimulus like a gentle air-puff. From previous genetic and cellular studies it is known that the IP_3_R in *Drosophila* is activated by G-protein coupled receptors (GPCRs). However, GPCRs that act upstream of the IP_3_R in the context of flight are not known. Therefore, we performed a genetic RNAi screen to identify GPCRs which regulate flight. This screen was followed by a secondary suppressor screen that assessed the role of each identified GPCR in activating IP_3_/Ca^2+^ signaling. We found 5 such GPCRs. Our results demonstrate that these GPCRs are required during flight circuit development and during adult flight. One flight-regulating receptor identified was the Pigment Dispersing Factor Receptor (PdfR). This receptor is known to regulate behaviors such as circadian rhythms, geotaxis and reproduction. A spatio-temporal analysis of PdfR flight function indicates that it regulates both flight circuit development and acute flight through multiple neurons. We postulate that PdfR signaling could modulate and integrate multiple behavioral inputs in *Drosophila* and other flying insects.

## Introduction

The evolution of flight in insects is linked to a number of natural behaviors including identifying food sources, mates and sites for egg-laying. The complexity of such behaviors frequently requires multiple sensory inputs that act directly and indirectly through neuromodulatory circuits, to control and fine-tune the final behavioral outcome [1], [2]. In the context of insect flight, the cellular and molecular bases of these neuromodulatory circuits are as yet ill-defined. Our interest in the flight circuit arose from the observation that mutations in the inositol 1,4,5-trisphosphate receptor (IP_3_R; *itpr*; [3], [4]), a ligand-gated Ca^2+^ channel that responds to IP_3_ generated after GPCR stimulation, resulted in strong flight deficits in *Drosophila*. These results suggested that G-protein coupled receptors (GPCRs) linked to IP_3_/Ca^2+^ signaling may play an important role in regulating flight behavior. While receptor tyrosine kinases can also initiate IP_3_/Ca^2+^ signaling in vertebrates, genetic evidence in *Drosophila* does not support this mode of IP_3_R activation [5].

The *Drosophila* genome contains ∼200 GPCRs, of which ∼90 have been identified as either gustatory or olfactory receptors [6], [7]; of the remaining GPCRs, although the ligands for most have been identified, the physiological function of only a small number is known. Some of the GPCRs are either identified or putatively assigned as receptors for neuropeptides that regulate feeding and foraging behavior, walking, modulation of visual processing and the response to stress [8]–[11]. Three neuropeptides (SIFamide, sex peptide and NPF) and their cognate receptors have been implicated in courtship behavior [12]–[14]. Recently, the receptors for DSK-1, DSK-2 and CCKLR-17D1 have been shown to regulate larval locomotion [15]. However, GPCRs that are involved in regulation of flight are still being discovered. Recent pharmacological evidence has implicated various monoamines such as octopamine, dopamine, tyramine and histamine (and presumably their receptors) and the muscarinic acetylcholine receptor (mAcR) in locust flight initiation [16]. The *Drosophila* mAcR increases IP_3_ dependent intracellular Ca^2+^ upon activation by its agonist in transfected S2 cells [17], [18] and in primary neuronal cultures from *Drosophila* [4]. *Drosophila* mutants that reduce octopamine levels exhibit flight initiation and maintenance defects which can be suppressed by pharmacological blocking of Tyramine receptors [19]. Signaling downstream of the Tyramine receptors suggests multiple mechanisms including cAMP [20], [21].

Here, we describe a genetic RNAi-based screen to identify GPCRs that regulate flight through IP_3_ mediated Ca^2+^ signaling. Among the GPCRs identified, two were previously known to activate IP_3_/Ca^2+^ signaling in neurons, but were not known to regulate flight in *Drosophila*. Furthermore, we show that GPCR signaling is required during development of the flight circuit as well as for modulation of adult flight. One of the GPCRs identified in our screen is the receptor for the Pigment Dispersing Factor or PdfR [22]. From analysis of PdfR expression in the nervous system in the context of flight and its well-defined roles in other behavioral paradigms, we propose that PdfR signaling functions systemically to integrate multiple sensory inputs and modulate downstream motor behavior.

## Results

### Identification of G-protein coupled receptors that modulate flight in *Drosophila*

**Figure 1:**
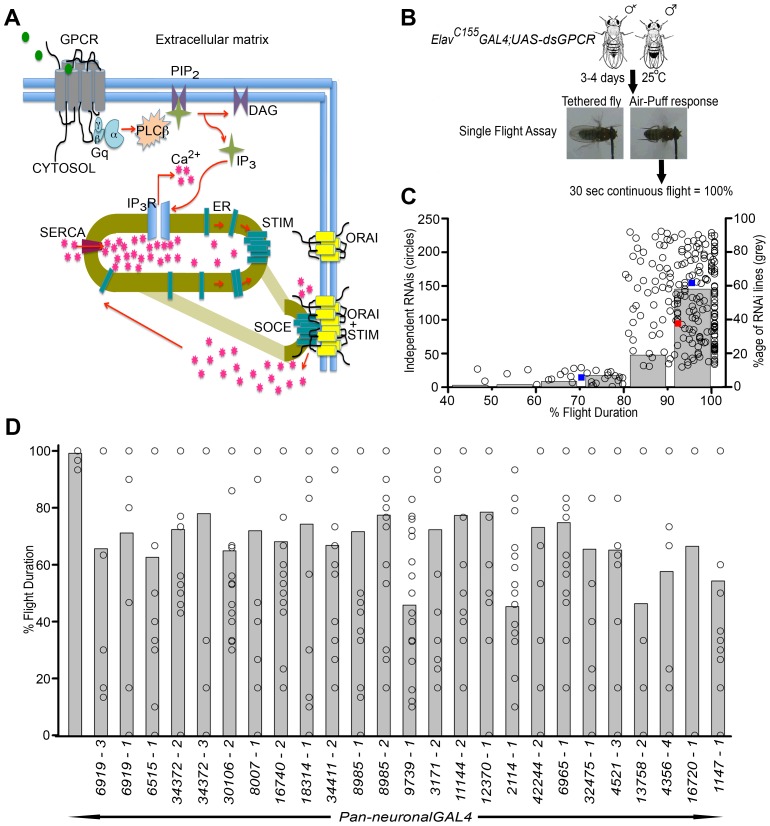
A genetic RNAi screen for G-protein coupled receptors that regulate flight in *Drosophila*. **A**) A schematic of how GPCR activation can stimulate the IP_3_R mediated Ca^2+^ signaling pathway and Store-operated Ca^2+^ entry (SOCE) through STIM and Orai. Gq is a heterotrimeric G-protein that acts downstream of IP_3_R linked GPCRs and activates PLCβ upon ligand-binding to the GPCR. STromal Interacting Molecule (encoded by *dSTIM* in *Drosophila*) is an ER membrane protein that can sense reduced Ca^2+^ in the ER store upon IP_3_R-mediated Ca^2+^ release and subsequently activates SOCE from plasma-membrane localized Orai channels. **B**) A schematic representation of the screening strategy for identifying G-protein coupled receptors (GPCRs) required in *Drosophila* flight. Flies (n≥10) with pan-neuronal (*Elav* ***^C155^*** *GAL4*) knockdown of individual GPCR were collected and tested for flight. Non-stop flight for 30 sec after a gentle air-puff was taken as 100% flight. **C**) Mean percentage time of flight for each genotype tested (open circles) is shown in increasing order. The average of all mean percentage flight times is shown as a red box. Average of mean percentage flight time for genotypes above and below 80% flight time are shown as blue boxes, and were found to be significantly different from each other (P<0.005). Therefore, flight time of 80% was considered as the significant cut-off for identifying putative GPCRs affecting flight. Grey bars show the number of RNAi strains lying within the indicated intervals of 10% of flight time. **D**) Individual GPCR RNAi strains identified with a mean percentage flight time of less than 80%. Each strain has been referred to by its CG number and the individual RNAi number (described in Table S1). Open circles within the bars show percentage flight times for each fly. Where flight times overlap a single open circle is shown. All RNAi heterozygotes (Table S1) and the pan-neuronal GAL4 used in these experiments (column on extreme left) showed normal flight durations.

To identify G-protein coupled receptors (GPCRs) that activate Gq-Plcβ signaling leading to IP_3_R mediated Ca^2+^ release (Figure 1A) during flight circuit development and function, an RNAi-based screen was designed with the *UAS-GAL4* system (Figure 1B). A total of 224 *UAS-RNAi* strains specific for 108 non-olfactory and non-gustatory GPCRs were selected based on a previous bioinformatic analysis ([6] and Table S1). Each of these RNAi strains were expressed individually using the pan-neuronal *Elav^C155^GAL4* strain which expresses in all post-mitotic neurons [23]. As indicated in the methods section, only adult female flies were tested for analysis of air-puff induced flight initiation and maintenance (Figure 1B). Normal initiation and maintenance of flight was observed upon pan-neuronal knockdown of 86 GPCRs (Figure 1C), while pan-neuronal knockdown of 22 GPCRs resulted in flight time of less than 80% (Figure 1D and Table S1). Flies with pan-neuronal knockdown of the 5HT1a receptor (*16720-2*), neuropeptide F receptor (*1147-2*), dromyosuppressin receptor 1 (*8985-4*) and methuselah-like 7 receptor (*7476-3*) showed wing posture (expanded wings) defects which affected their flight ability (Figure S1); wing-posture defects however were not uniform, with a small fraction exhibiting normal wings and flight (data not shown). Pan-neuronal knockdown of other methuselah-like receptors such as the methuselah-like 8 receptor (*32475-2*), methuselah-like 9 receptor (*17084-3*) and methuselah-like 6 receptor (*16992-3*) showed similar expanded wing phenotypes in a fraction of the animals. Flies with normal wings also showed normal flight ability (data not shown). Pan-neuronal knockdown of the SiFamide receptor (*10823-1, SiFaR*) resulted in lethality during pupal stages (see later). Therefore, our screen at this stage yielded 22 putative GPCRs whose function appeared to be required for maintenance of air-puff induced flight in *Drosophila*.

### Over-expression of the endoplasmic reticulum store Ca^2+^ sensor *dSTIM* and a constitutively active form of *dgq* (*AcGq*) rescues flight defects by pan-neuronal knockdown of the IP_3_R (*itpr*)

**Figure 2:**
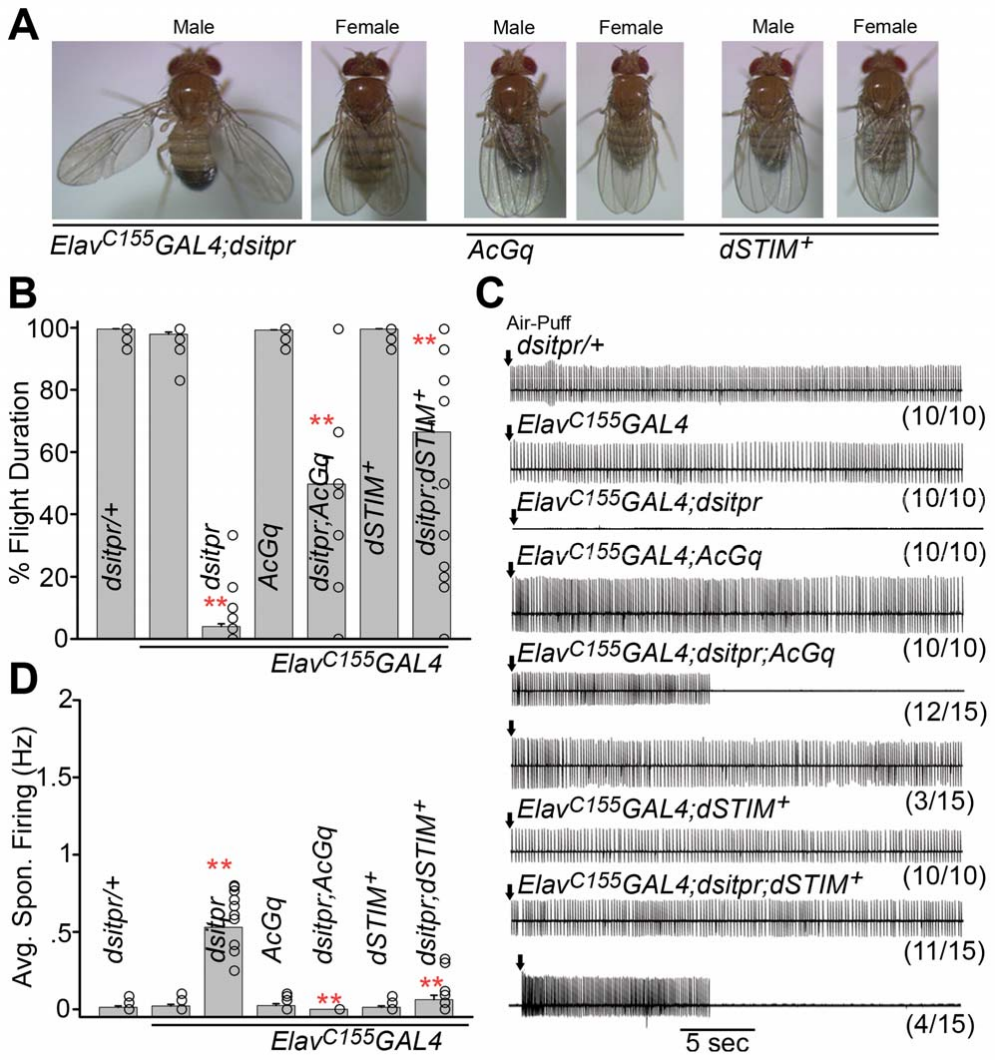
Flight-deficits in flies with pan-neuronal knockdown of the IP_3_R can be rescued by expression of *AcGq* and *dSTIM^+^*. **A**) Pan-neuronal expression of either *AcGq* or *dSTIM^+^* suppresses wing posture defects in flies with pan-neuronal knockdown of the IP_3_R (*dsitpr; dicer*). All 100 flies analyzed showed normal wing posture. **B**) Air-puff stimulated tethered flight was compromised by pan-neuronal knockdown of the IP_3_R. Significant rescue of the mean percentage flight time was observed by expression of either *AcGq* or *dSTIM^+^*. Results are expressed as mean ± SEM of the flight time of 30 flies tested individually for flight. Open circles within the bars show the percentage flight times for individual flies. Pan-neuronal knockdown of the IP_3_R was compared with the pan-neuronal GAL4 control; and *AcGq* and *dSTIM* ^+^ rescues were compared to the *itpr* knockdown (one-way ANOVA, **P<0.01). **C**) Electrophysiological recordings from the DLMs of air-puff stimulated (arrows) tethered flies are shown. The genotypes are indicated above the traces, and the numbers indicate the number of flies with the observed flight pattern over the number of flies tested. All control flies show rhythmic firing throughout flight. Complete loss of electrical activity was seen in flies with pan-neuronal expression of *dsitpr*. Expression of either *AcGq* with *dsitpr* (*Elav^C155^GAL4; dsitpr; AcGq*) or *dSTIM^+^* with *dsitpr* (*Elav^C155^GAL4; dsitpr; dSTIM^+^*) restored electrical firing from the DLMs to varying extents, which is shown as two categories below the indicated genotype. **D**) Quantification of spontaneous firing from DLMs of the indicated genotypes. Average spontaneous firing was restored to normal upon expression of *AcGq* or *dSTIM^+^* in pan-neuronal knockdown of IP_3_R. Results are shown as mean ± SEM. Open circles within the bars are the average spontaneous firing quantified for individual flies. Pan-neuronal knockdown of the IP_3_R (*dsitpr*) was compared to pan-neuronal GAL4 controls and rescues were compared to the knockdown (**P<0.01, one-way ANOVA).

Previous studies have shown that pan-neuronal knockdown of the IP_3_R with an inducible RNAi leads to significant defects in wing posture and flight ([24]; Figure 2A and 2B). To identify GPCRs that stimulate IP_3_ mediated Ca^2+^ release a secondary suppressor screen was devised and tested as follows: *dgq* codes for the alpha subunit of the heterotrimeric G-protein, Gq and activates phospholipase Cβ upon binding of the cognate ligand to the GPCR (Figure 1A). Genetic interactions in the context of *Drosophila* flight have been demonstrated previously between *dgq* and *itpr* mutants [5]. *dSTIM* codes for the *Drosophila* STromal Interaction Molecule (dSTIM) which functions as a sensor of endoplasmic reticulum (ER) store Ca^2+^ [25], [26]. Depletion of ER Ca^2+^ activates STIM followed by opening of the Orai (*dOrai*) surface channel, also referred to as the store-operated Ca^2+^ entry (SOCE) channel. Previous observations with *itpr* mutants support the idea that STIM and Orai function with the Sarco-Endoplasmic Reticulum Ca^2+^ ATPase pump (SERCA) to restore Ca^2+^ levels in the ER lumen of *Drosophila* neurons after GPCR activation and IP_3_-mediated Ca^2+^ release [4], [5], 24. Therefore pan-neuronal expression of either a constitutively activated form of Gq (*Gq^Q203L^* or *AcGq*; [27]) or *dSTIM^+^* were first tested for their ability to suppress flight deficits in flies with pan-neuronal knockdown of the IP_3_R, using a previously validated *itpr* RNAi strain (*dsitpr*; [24]). Pan-neuronal knockdown of the IP_3_R leads to a near complete flight deficit (4%±1.94). While *dSTIM^+^* over-expression could suppress this loss of flight and restore it up to 65%, in *AcGq* expressing animals the flight deficit was restored to 50% (Figure 2B). Physiological correlates of flight, such as electrophysiological recordings from the dorsal longitudinal muscles (DLMs) of *dsitpr; dSTIM^+^* and *dsitpr; AcGq* expressing flies showed that 11/15 flies flew normally and 4/15 flies flew for 15 sec with *dSTIM^+^* while 3/15 flies flew normally and 12/15 flies flew for 10–15 sec with *AcGq* (Figure 2C). Wing posture defects and spontaneous firing from the DLMs observed in flies with pan-neuronal knockdown of the IP_3_R were rescued in all flies by expressing either *AcGq* or *dSTIM^+^* (Figure 2A and 2D). Thus, reduced signaling through the IP_3_R in *Drosophila* flight circuit neurons can be restored significantly either by increasing the active form of Gq (*AcGq*) or by raising SOCE through over-expression of *dSTIM* ^+^.

### Identification of GPCRs coupled to IP_3_ signaling and required for maintenance of flight in *Drosophila*

**Figure 3:**
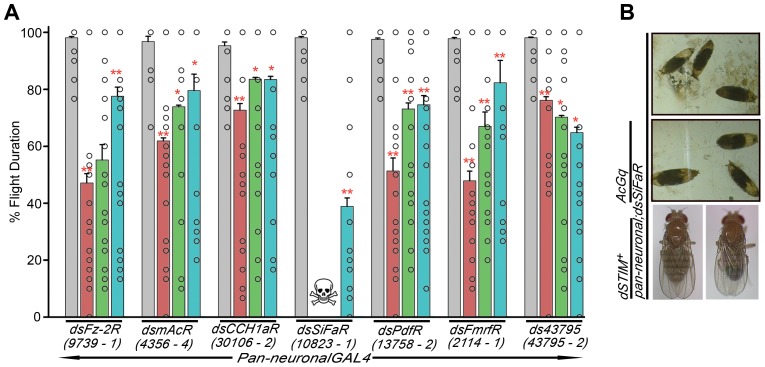
G-protein coupled receptors that regulate flight in *Drosophila* through IP_3_R mediated Ca^2+^ signaling. **A**) Percentage flight times are shown. The bars represent RNAi heterozygotes (grey), pan-neuronal RNAi knockdown (red), pan-neuronal RNAi knockdown plus *AcGq* (green) and pan-neuronal knockdown plus *dSTIM^+^* (blue). Mean flight times (± SEM) were obtained by measuring tethered flight in three batches of 10 tethered flies of each genotype after an air-puff stimulus. Open circles within the bars indicate percentage flight times for each fly. Pan-neuronal knockdown of GPCRs (red) was compared to pan-neuronal GAL4 controls (grey) and the rescues (blue, green) were compared to the knockdown (red; *P<0.05, one-way ANOVA). **B**) Pan-neuronal knockdown of the SiFamide receptor (*10823-1*) results in pupal lethality (shown as a skull in A). Lethality was suppressed by pan-neuronal over-expression of *dSTIM^+^* but not *AcGq*.

GPCRs linked to IP_3_R mediated Ca^2+^ signaling and required for the maintenance of flight were identified from amongst the 22 receptors shown in Figure 1D by individual pan-neuronal GPCR knockdowns in the context of over-expression of *AcGq* and *dSTIM^+^* transgenes. The resulting progeny were tested in the single flight assay (Figure 3A and S2). Out of the 22 putative receptors, flight was rescued to a significant extent for 4 receptors, namely mAcR (*CG4356*), CCH1aR (*CG30106*), PdfR (*CG13758*) and FmrfR (*CG2114*), by over-expression of either *dSTIM^+^* or *AcGq* or both (Figure 3A). Therefore, our screen identified mAcR, CCH1aR, PdfR and FmrfR as the GPCRs that are required for the maintenance of *Drosophila* flight through IP_3_ mediated Ca^2+^ signaling.

From the remaining 18 receptors, flight defects for the frizzled-2 receptor (*CG9739* or *dFz-2R*) were suppressed to a significant level by expression of *dSTIM^+^*. Interestingly, *AcGq* expression did not have a significant effect in flies with pan-neuronal knockdown of *dFz-2R* (Figure 3A). In flies with knockdown of *CG43795* (two independent RNAi constructs: *43795-1*, *43795-2*), rhodopsin-like receptor (*16740-2*), a neuropeptide receptor (*34411-2*), trapped in endoderm (*3171-2*) and diuretic hormone 44 receptor 2 (*12370-1*), flight time reduced further upon pan-neuronal expression of either *AcGq* or *dSTIM^+^* or both (Figure S2). Flight time was reduced significantly by both in *34411-2*, *3171-2* and *43795-2* (Figure S2 and 3A). However, only *43795-2* was investigated further (see discussion).

Pupal lethality was observed upon knockdown of the SiFamide receptor (*10823-1; SiFaR*) in neurons (Figure 3A and 3B) which could be rescued completely by pan-neuronal expression of *dSTIM* ^+^. Interestingly, there was no rescue of lethality by *AcGq* (Figure 3B). Adult flies that eclosed after over-expressing *dSTIM^+^* in background of SiFamide receptor down-regulation had normal wings, but showed significantly reduced flight time (40%; Figure 3A).

While the validation by *AcGq* and *dSTIM^+^* expression helped confirm signaling through the IP_3_R in the case of receptors shown in Figure 3A, it was of concern that in each case just one RNAi line for each GPCR showed flight deficits. We therefore tested the efficiency of GPCR knockdown for each RNAi strain, validated by rescue with either *AcGq* or *dSTIM^+^*, in a qPCR analysis. Two RNAi lines were selected for each validated GPCR; one that gave a flight defect and another that did not. The transcript level for each GPCR was quantified from isolated larval brains with pan-neuronal knockdown of the GPCR in the two selected RNAi lines. In all cases RNA levels were reduced to approximately half of wild-type in RNAi strains that showed flight deficits, but not in cases where flight was maintained for normal periods (Figure S3). Thus, differential efficacy of RNAi strains appears to be responsible for the absence of flight deficits by multiple RNAi lines for a particular GPCR.

### IP_3_R function is primarily required from 16 to 32 hours after puparium formation to regulate flight

**Figure 4:**
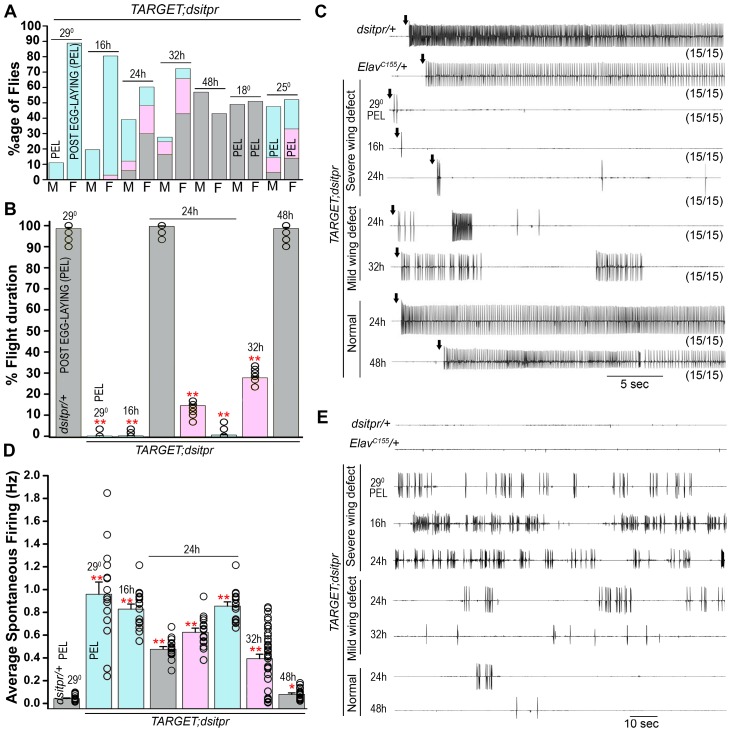
IP_3_R function is required from 16 to 32 hours after puparium formation to regulate flight. **A**) Quantification of wing posture defects by knockdown of the IP_3_R (*dsitpr*) at specific stages during pupal development indicated above the bars using the *GAL4/GAL80ts* system (*TARGET*), are shown. Mixed populations of normal (grey bar), mild wing posture defect (pink bar) and severe wing posture defective (blue bar) flies were obtained when *dsitpr* is expressed from 16 h, 24 h or 32 h after puparium formation (APF) to adulthood, but not when expression is induced at 48 h APF and onwards. Animals were either moved from 18°C to 29°C, at the appropriate pupal phases to induce RNAi expression or maintained at a constant temperature post egg-laying (PEL) as indicated. Percentage of animals with varying wing posture defects is shown as a stacked histogram for males (M) and females (F). **B**) Quantification of flight duration in single flight assays (mean ± SEM) from female animals of the indicated genotypes. Open circles within each bar represent percentage flight time for each fly. Mean flight times (± SEM) were obtained for three batches of 10 tethered flies of each genotype after an air-puff stimulus. All the knockdowns were compared to 29°C *dsitpr/+* control (**p<0.01, one-way ANOVA). Color codes of the histograms are the same as in (A) above. **C**) Representative traces of electrophysiological recordings from DLMs of the indicated genotypes in response to a manual air-puff stimulus (arrow). The number of flies that showed the given response upon the number of flies tested is given below each trace. Adult female flies were sorted on the basis of the severity of their wing defects as shown. Loss of rhythmic flight patterns accompanied by severe wing posture defects is observed due to pan-neuronal depletion of IP_3_R during early pupal development (16 h APF). Knockdown at later time points shows reduced flight duration in flies with mild wing posture defect and normal flight in animals with normal wings. **D**) Quantification of the frequency of spontaneous firing as recorded from DLMs of the indicated genotypes. As with flight deficits and air-puff induced electrical firing patterns, spontaneous firing frequencies are higher in animals with wing posture defects (color coded as in A) and the time of transfer to 29°C. (**p<0.01, *p<0.05, one-way ANOVA, N = 15). Firing frequencies were calculated by counting the number of spikes over 2 min. Individual data points are represented by open circles. **E**) Representative traces for (D) showing increased spontaneous firing in animals with severe wing posture defect due to early knockdown of the IP_3_R.

Expression of *itpr^+^* between 16 to 48 hours after puparium formation (APF) is sufficient for rescue of adult flight in *itpr* mutants, suggesting that a major role of IP_3_-mediated Ca^2+^ release in the flight circuit maybe during development [3]. Before testing if requirement for the identified GPCRs was during pupal development or in adult flight, we sought to characterize the time window (16–48 hr APF) for *itpr* requirement more closely. For this purpose we used the TARGET (temporal and regional gene expression targeting) system [28] which includes a temperature sensitive GAL80 element (*GAL80^ts^*) that regulates GAL4 in a temperature dependent manner, with optimal repression and expression of GAL4 observed at 18°C and at 29°C respectively [29]. Experimental animals of the genotype *Elav^C155^GAL4/+; dsitpr/+; GAL80^ts^/+* were shifted to the permissive temperature (29°C) at specific time points after puparium formation (APF). This allowed expression of the IP_3_R RNAi (*dsitpr*) and down-regulation of *itpr* transcripts from the time point of the temperature shift. Flies with a range of wing posture defects were observed upon pan-neuronal knockdown of the IP_3_R at 16 hours, 24 hours and 32 hours APF (Figure 4A). Moreover, from the ratio of males and females obtained, there is an apparent lethality in males at the permissive temperature (29°C). The occurrence of a more severe defect in males as compared to females is very likely due to sex-specific differences in expression of the *Elav^C155^GAL4* transgene, which is inserted on the X chromosome. Adults that emerged from these time points were quantified for the severity of wing posture defects (Figure 4A). These were correlated with their ability to sustain flight (Figure 4B). A strong correlation was observed between the ability to fly and the extent of wing posture defects in animals from all time points. Pan-neuronal knockdown of *itpr* starting at 16 hours APF lead to a complete loss of flight as evident from the single flight assay (Figure 4B) and air-puff induced flight patterns recorded from the DLMs (Figure 4C). These animals also exhibited a significant level of spontaneous firing activity (SPF) from the DLMs, which is characteristic of *itpr* mutants (Figure 4D and 4E; [3]). Animals with knockdowns at later stages showed a range of flight deficits that correlated well with their observed wing posture deficit and recordings from the DLMs (Figure 4A–E), though SPF was high in all flies from the 16 hours and 24 hours APF time points, regardless of wing posture. When the temperature shift to 29°C was made 48 hours APF or later, neither wing posture nor flight deficits were observed (Figure 4A–E; data not shown for 96 hours and 144 hours APF). IP_3_R is thus necessary from 16–32 hours APF for normal flight circuit development.

### Identification of GPCRs required during pupal development

**Figure 5:**
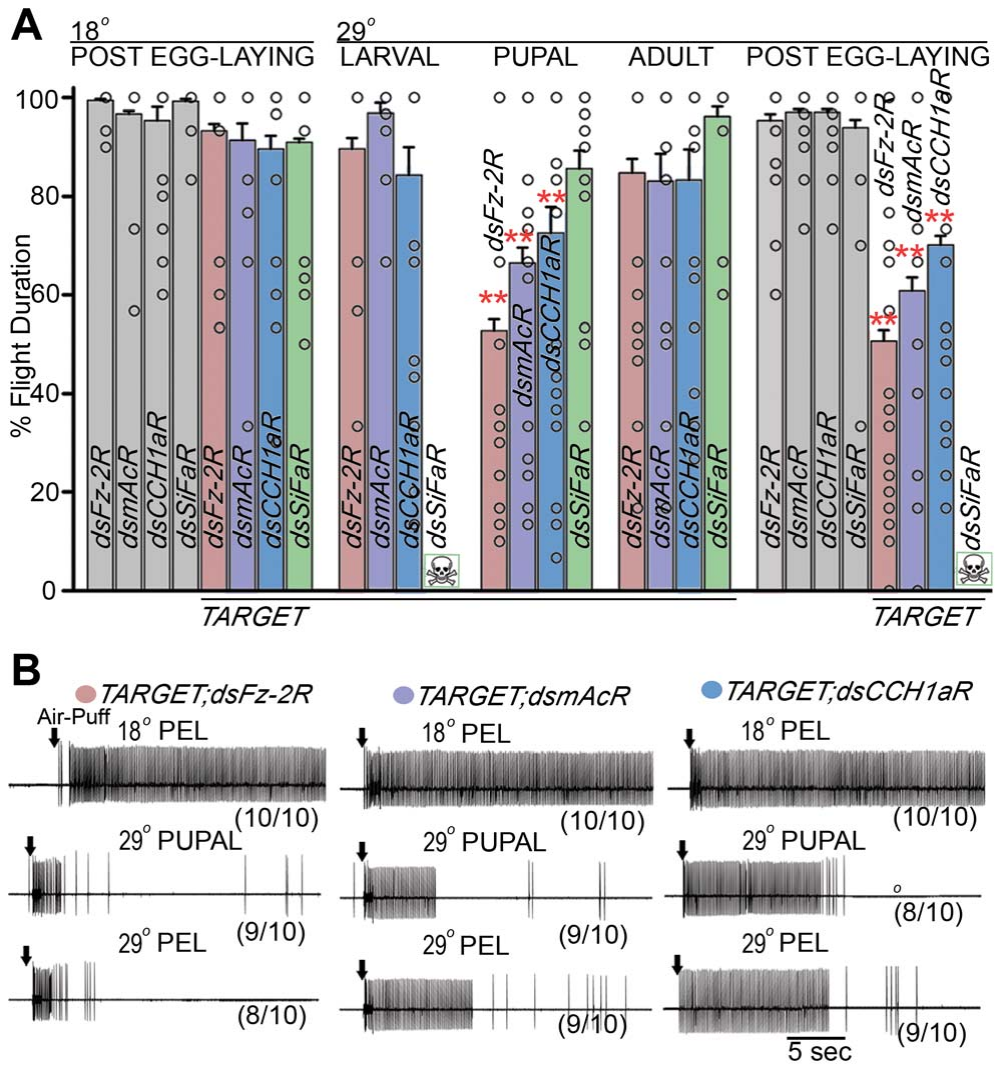
Adult flight deficits result from RNAi knockdown of specific GPCRs during pupal development. **A**) Percentage flight time of RNAi heterozygotes (grey bars) and pan-neuronal knockdown of GPCR RNAi strains (*dsFz-2R* in red, *dsmAcR* in purple, *dsCCH1aR* in blue, *dsSIFaR* in green) at specific developmental stages, as indicated above the bars using the *GAL4/GAL80ts* system (*TARGET*), are shown. Open circles within each bar represent percentage flight time for each fly. Mean flight times (± SEM) were obtained for three batches of 10 tethered flies of each genotype after an air-puff stimulus. Pupal knockdowns at 29°C were compared with the specific RNAi control at 18°C; post egg-laying (PEL) knockdowns were compared with their specific RNAi controls (**P<0.01, *P<0.05; one-way ANOVA). **B**) Electrophysiological recordings from the DLMs of air-puff stimulated tethered flies with pan-neuronal knockdown of RNAi (*dsFz-2R* in red, *dsmAcR* in purple, *dsCCH1aR* in blue). The temporal stage at which knockdown was initiated is indicated above each trace. Numbers in brackets below each trace is the number of flies with the given response upon the number of flies tested. The remaining flies in each case showed a normal firing response. All flies maintained at 18°C show rhythmic firing throughout flight. Flight pattern durations were reduced in flies either with pupal knockdowns (PUPAL 29°C) or kept at 29°C post egg-laying (PEL).

Next, we investigated the temporal requirement for the identified GPCRs in the context of flight. These experiments demonstrated that pan-neuronal knockdown of either *dFz-2R*, *mAcR* or *CCH1aR* during pupal stages leads to flight deficits in adults, when tested in single flight assays (Figure 5A). Similarly treated RNAi heterozygotes resulted in normal flight (data not shown). The percentage of flight time was reduced upon pupal knockdown of *dFz-2R* to 53%±2, *mAcR* to 66%±3 and *CCH1aR* to 72%±5 (Figure 5A, colored bars within 29°C pupal, Movie S1). Air puff stimulated responses recorded from DLMs were absent in a majority of non-fliers selected after the single flight assay, by pupal knockdown of *dFz-2R* (9/10), *mAcR* (9/10) or *CCH1aR* (8/10; Figure 5B). Importantly, knockdown of *dFz-2R*, *mAcR* and *CCH1aR* during pupal stages resulted in flight deficits for each receptor that were similar to the deficits observed by knockdown throughout development (shifted to 29°C post egg-laying) (Figure 5A and 5B), indicating that the requirement for all three GPCRs is primarily during flight circuit development. Similar experiments of *SiFaR* knockdown demonstrated a vital requirement during larval stages which lead to pupal lethality (Figure 5A, green). However, knockdown of *SiFaR* during pupal stages did not affect flight duration indicating that this GPCR does not have a measurable role in either flight circuit development or in regulating flight in adults (Figure 5A).

### Identification of GPCRs required both during development and in adults

**Figure 6:**
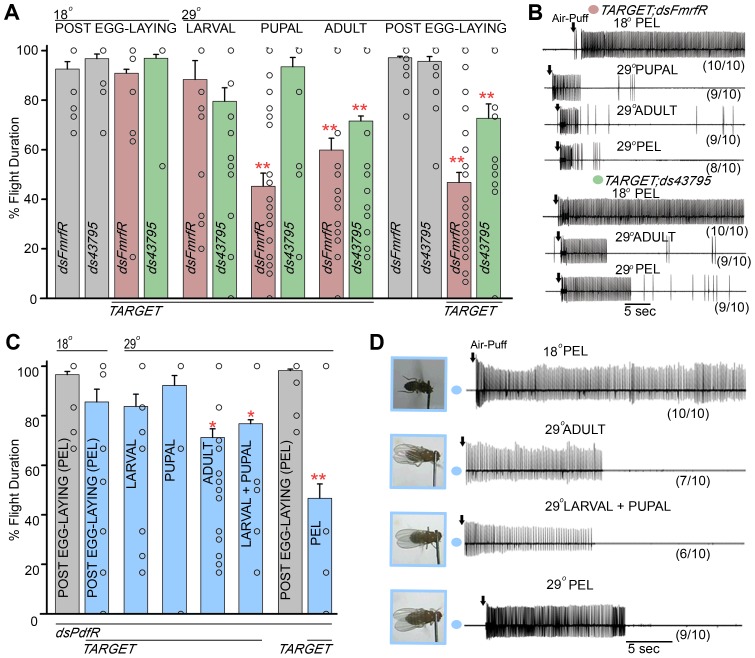
RNAi mediated knockdown of specific GPCRs during development and in adults result in flight deficits. **A**) Percentage flight times of GPCR RNAi heterozygotes (grey) and pan-neuronal knockdowns of RNAi strains (*dsFmrfR* in red, *ds43795* in green) were obtained after knockdown from specific developmental stages as indicated above the bars. Open circles within the bars represent percentage flight times for individual flies. Mean flight times (± SEM) were obtained by measuring tethered flight in three batches of 10 tethered flies of each genotype after an air-puff stimulus. Pupal and adult knockdowns at 29°C were compared with the specific RNAi control at 18°C; post egg-laying (PEL) knockdowns were compared with their specific RNAi controls at 29°C (**P<0.01, *P<0.05; one-way ANOVA). **B**) Electrophysiological recordings from the DLMs of air-puff stimulated tethered flies of the indicated genotypes. The temporal stages at which knockdowns were initiated are indicated above each trace. Also shown below each trace is the number of flies that showed the given response upon the number of flies tested. In each case the remaining flies showed normal firing responses. All control flies at 18°C showed rhythmic firing throughout flight. **C**) Percentage flight time (mean ± SEM) of flies with pan-neuronal knockdown of the *PdfR*. Knockdown was initiated from different developmental stages at 29°C as indicated. Open circles within the bars show percentage flight times for each fly. Pan-neuronal knockdown of the *PdfR*, either in adults or during development (larval + pupal), results in significant flight deficits (*P<0.05, as compared to 18°C *PdfR* knockdown control, by one way ANOVA). Strongest flight deficits are observed upon *PdfR* knockdown throughout development by shifting to 29°C post egg-laying (PEL; **P<0.01, when compared to RNAi heterozygotes at 29°C post egg-laying). **D**) Snapshots of single flight assay video recordings and electrophysiological recordings from DLMs of animals with pan-neuronal knockdown of the *PdfR* at the indicated temperatures and developmental stages. The number of flies that showed the given response upon the number of flies tested is shown below each trace. The remaining animals showed normal flight and firing responses as seen in the 18°C PEL control on top.

Next, temporal requirements for the *FmrfR* and *CG43795* were investigated using similar TARGET based experiments as described for the previous set of GPCRs in Figure 5. Interestingly, knockdown of *FmrfR* either in adults or during pupal development resulted in flight deficits (Figure 6A, red bars, Movie S2). The extent of flight deficits by pan-neuronal knockdown of the *FmrfR* at the pupal stage was 45%±5, while at the adult stage it was 60%±5 (Figure 6A; red bars under 29°C pupal and 29°C adult). These deficits are comparable with post egg-laying (PEL) knockdown of the *FmrfR*, maintained all through development (47%±2; Figure 6A, red bar under 29°C post egg-laying). Similarly treated RNAi heterozygotes resulted in normal flight (data not shown). Air puff stimulated responses obtained by electrophysiological recordings from the DLMs of the non-fliers selected after single flight assays were absent in 9/10 flies during pupal knockdown and in 9/10 flies during adult knockdown of the *FmrfR* (Figure 6B). These deficits are comparable qualitatively and quantitatively with recordings from non-fliers obtained after down-regulation of *FmrfR* throughout development, where 8/10 animals exhibited rhythmic flight patterns for 5 sec or less (Figure 6B, 29°C PEL). Thus the *FmrfR* receptor is required for modulation of flight both during pupal development and acute flight in adults.

Unlike all other GPCRs identified in this screen, the requirement for *CG43795* was only at the adult stage. Flies with adult knockdown of *CG43795* showed reduced flight with a percentage flight time of 72%±2 (Figure 6A, green bar below 29°C adult). Electrophysiological recordings from the DLMs of *CG43795* knockdown non-fliers showed loss of flight patterns, upon air-puff stimulation, after 10–12 sec in 9/10 flies (Figure 6B, 29°C adult). These flight deficits were comparable with the deficits observed upon knockdown of *CG43795* throughout development (Figure 6A, green bar in 29°C post egg-laying and 6B, 29°C PEL).

Next, temporal requirement for the *PdfR* was assessed by similar TARGET based experiments. While the expression of *dsPdfR* during either larval or pupal stages had no significant effect on flight (Figure 6C, blue bars within 29°C larval and 29°C pupal), its knockdown through both (larval and pupal) stages of development resulted in significant reduction in flight time (77%±2; Figure 6C, blue bar within 29°C larval+pupal) and was accompanied by shorter periods of air-puff induced rhythmic action potentials recorded from the DLMs (Figure 6D, 29°C larval+pupal). In addition, significant flight deficits and associated changes in flight physiology were observed upon *PdfR* knockdown in adults (Figure 6C and 6D, blue bar and trace within 29°C adult). The flight deficit obtained by *PdfR* knockdown in larval and pupal development (77%±2) and by adult knockdown (71%±3), together recapitulates the flight deficit observed when the *PdfR* RNAi was expressed throughout development (shifted to 29°C post egg-laying; 54%±6; Figure 6C). These data suggest that signaling through the PdfR is required in separate neuronal subsets through development and in adults, and that both subsets contribute additively to the complete flight phenotype observed by PdfR knockdown through development and in adults.

### Intracellular Ca^2+^ and PdfR signaling are required in the same neuronal domain of *PdfR* expression for flight

**Figure 7:**
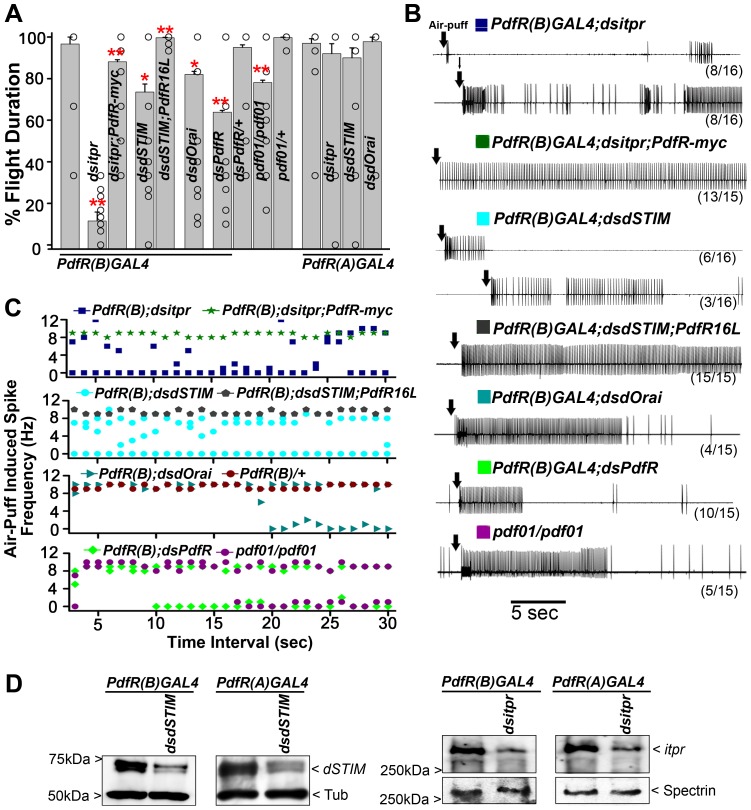
Depletion of IP_3_R, SOCE and PdfR in a specific sub-domain of PdfR expressing neurons affects flight duration. **A**) Significant flight deficits were obtained upon knockdown of the IP_3_R (*dsitpr*), the SOCE components, *dSTIM (dsdSTIM)* and *dOrai* (*dsdOrai*) by the *PdfR(B)GAL4* strain (**P<0.01 and *P<0.05; compared to *PdfR(B)GAL4* heterozygote controls). Flight deficits induced by *dsitpr* in *PdfR(B)GAL4* expressing neurons were rescued by the *PdfR-myc* genomic construct (**P<0.01 compared to *dsitpr* knockdown). Similarly, the flight phenotype in *PdfR(B)GAL4;dsdSTIM* animals could be rescued by over-expression of *PdfR* (*UAS-PdfR16L*; **P<0.01 as compared to *dsdSTIM* knockdown). Knockdown of the *PdfR* in *PdfR(B)GAL4* expressing neurons also resulted in significant flight deficits (**P<0.05; *dsPdfR/PdfR(B)GAL4* compared with *dsPdfR*/+). Flight duration in the PDF null allele (*pdf01/pdf01*) was significantly reduced as compared with *pdf01* heterozygotes (**P<0.01). All significance values were obtained by one-way ANOVA tests. No flight defects were observed upon knockdown of the IP_3_R (*dsitpr*), *dSTIM (dsdSTIM)* and *dOrai (dsdOrai)* in *PdfR(A)GAL4* expressing cells. **B**) Representative electrophysiological recordings from DLMs of tethered flies are shown after an air-puff stimulus (arrows). Genotypes are indicated above the traces. Shown below each trace is the number of flies in which the given response was elicited from amongst the total number of flies tested. In each case normal patterns and durations were observed in the remaining flies (not shown). Significant loss of rhythmic flight patterns were observed upon knockdown of the IP_3_R (dark blue) shown in two categories, *dSTIM* (light blue) shown in two categories, *dOrai* (dark cyan) and *PdfR* (light green) in the *PdfR(B)GAL4* domain. Normal firing patterns were restored by expression of *PdfR-myc* (green) and *PdfR16L* (dark grey) in flies with IP_3_R (*dsitpr*) and *dSTIM* knockdowns respectively. PDF null mutants (*pdf01/pdf01*; magenta) also showed a reduced duration of rhythmic firing patterns. *PdfR(B)GAL4* heterozygotes and RNAi heterozygotes showed rhythmic firing throughout flight (data not shown). **C**) Quantification of the spike frequency for electrophysiological traces (as shown in C) for the indicated genotypes. **D**) Western blots from protein extracts of adult brains and thoracic ganglia. Expression of *dSTIM* and the IP_3_R is reduced, upon their knockdown with specific RNAi expression in *PdfR(B)GAL4(2)* and *PdfR(A)GAL4(2)* strains.

To identify *PdfR* expressing neurons which require Ca^2+^ release through the IP_3_R and SOCE for maintenance of flight, five independent GAL4 constructs that drive expression in PdfR neurons were tested [30]. These GAL4 constructs contain different regions of the PdfR regulatory domain and thus essentially drive expression in subsets of PdfR neurons [30]. The five GAL4s were used to knockdown either *itpr*, *dSTIM* or *dOrai*. Flies with knockdown of the IP_3_R using *PdfR(B)GAL4* exhibited strong flight deficits (11%±3; Figure 7A) and wing posture defects in 10% males (data not shown). Moreover, air puff stimulated responses from the DLMs were found to be reduced and arrhythmic. In 8/16 animals, there was near complete loss of firing while 8/16 flies showed arrhythmic firing patterns (Figure 7B and 7C, navy blue). Importantly, wing posture defects, flight defects and reduced response from the DLMs of *PdfR(B)GAL4;dsitpr* organisms could be rescued by introducing a genomic construct for the *PdfR* referred to as *PdfR-myc* (Figure 7A–C; [30]).

Flight deficits were also observed upon reduction of SOCE in *PdfR(B)GAL4* expressing neurons either by knockdown of *dSTIM* (27%±3) or *dOrai* (18%±1; Figure 7A). Knockdown of *dSTIM* resulted in reduced firing from DLMs in 6/16 flies (∼5 sec) and arrhythmic firing in 3/16 flies (Figure 7B and 7C, light blue). Knockdown of *dOrai*, showed reduced firing in just 4/15 flies (∼15 sec; Figure 7B and 7C, green). Knockdown of d*STIM* using *PdfR(B)GAL4* also resulted in increased spontaneous firing from the DLMs (data not shown); this phenotype is characteristic of *itpr* mutants [3]. Importantly, all flight phenotypes including reduced electrophysiological responses from DLMs and the high spontaneous firing observed in *dSTIM* knockdown flies could be rescued to normal levels by over-expression of an inducible *PdfR* cDNA (*UAS-PdfR16L*; Figure 7A–C).

Next, we investigated the requirement for the PdfR directly in the *PdfR(B)GAL4* expressing neurons in the context of flight. Knockdown of PdfR (*dsPdfR*) in neurons marked by the *PdfR(B)GAL4* resulted in significant reduction in flight time (63%±0.8; Figure 7A) and in firing responses from the DLMs in 10/15 flies (∼5 sec; Figure 7B and 7C). Further, we investigated if mutants in the cognate ligand for the PdfR, the “Pigment Dispersal Factor” (*pdf*) affected flight. We tested adults for the null allele, *pdf01* for flight [31]. Homozygous *pdf01* showed reduced flight time (78%±1; Figure 7A) and reduced firing from DLMs in 5/15 randomly selected flies (Figure 7B and 7C). However, the flight defects observed either by knockdown of *PdfR* or in *pdf* mutant flies, were not equivalent to the deficits observed by knockdown of IP_3_R using *PdfR(B)GAL4* (Figure 7A). These data suggest that whereas PDF activates the PdfR in the *PdfR(B)GAL4* expressing neurons, there are probably additional roles for the IP_3_R in *PdfR(B)GAL4* expressing neurons in the context of flight. Furthermore, the flight deficits observed in PDF mutant flies (Figure 7A) were considerably less than flight deficits observed by knockdown of *PdfR* using *Elav^C155^GAL4* (Figure 3A), suggesting the existence of another flight-regulating ligand acting through the *PdfR*.

**Figure 8:**
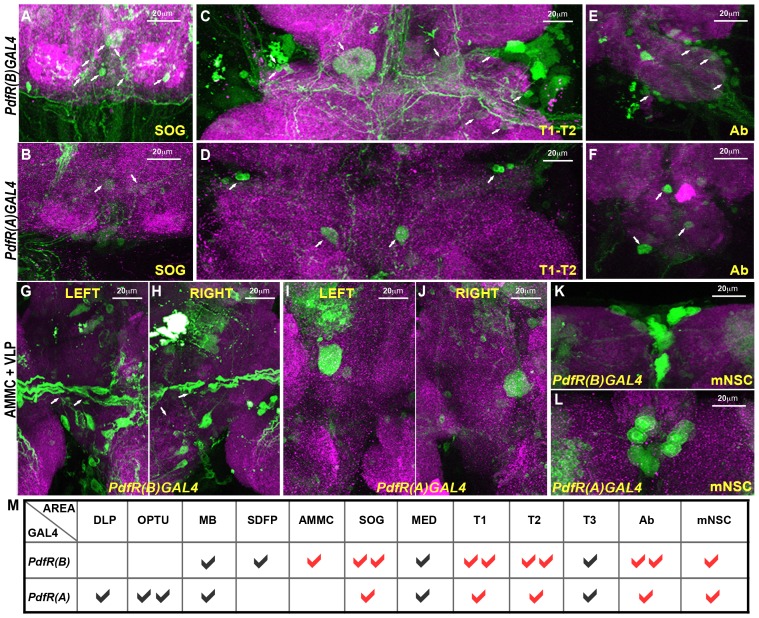
Differential expression of *PdfRGAL4* strains in neurons of the adult AMMC, SOG and thoracic ganglia. Expression patterns of the indicated *PdfRGAL4* strains in the sub-esophageal ganglion, SOG (A, B), thoracic region 1 and 2, T1–T2 (C, D) and abdominal regions, Ab (E, F) are shown. A schematic of these regions of the *Drosophila* central nervous system is given in Figure S4. GFP expression is reduced in the SOG, TI, T2 and abdominal region in *PdfR(A)GAL4*. Expression of GFP in left (G, I) and right (H, J) ventrolateral protocerebrum (VLP) and antennal mechanosensory and motor complex (AMMC) using *PdfR(B)GAL4* (G, H) and *PdfR(A)GAL4* (I, J) driver is shown. White arrows indicate neuron/cluster of neurons which are positive for GFP expression. GFP expression is reduced in the AMMC when using *PdfR(A)GAL4* driver as compared to *PdfR(B)GAL4*. Expression pattern of GFP in medial neurosecretory region, mNSC (K, L) using *PdfR(B)GAL4* (K) and *PdfR(A)GAL4* (L) drivers are shown. GFP expression is similar in medial neurosecretory region in *PdfR(B)GAL4* and *PdfR(A)GAL4*. M) Summary table of expression patterns of *PdfR(B)GAL4* and *PdfR(A)GAL4*. The areas that were examined: DLP: dorsolateral protocerebrum, OPTU: optic tubercle, MB: mushroom body, SDFP: superior dorsofrontal protocerebrum, MED: medulla. Ticks indicate the presence of expression. Two ticks indicate high expression. Red ticks represent areas that have been shown in high magnification. For each genotype, 5 brain samples were analyzed.

Knockdown of the IP_3_ receptor, dSTIM or dOrai using an independent transgenic line, the *PdfR(A)GAL4* [30], had no effect on normal wing posture (data not shown) or flight (Figure 7A). However, expression of dSTIM and IP_3_R was reduced significantly in adult brain and thoracic ganglia upon knockdown by RNAi using both the *PdfR(B)GAL4* and *PdfR(A)GAL4* (Figure 7D). These data suggest that the PdfR regulates flight through IP_3_R mediated Ca^2+^ signaling exclusively in the neurons marked by the *PdfR(B)GAL4* and not the *PdfR(A)GAL4*. Thus, to identify neuronal regions that require PdfR mediated Ca^2+^ signaling for regulation of flight, we compared neurons marked by expression of the *PdfR(B)GAL4* and *PdfR(A)GAL4*. For this purpose cells in both GAL4 strains were marked with a cytosolic form of GFP. The overall expression level of GFP in adult brains and ventral ganglia were similar in both the GAL4 strains (Figure S4C). Expression patterns of each GAL4 line were visualized in the larval brain, the adult brain and the thoracic ganglion (Figure S4A, S4B, S4E and S4F). Expression patterns were analyzed by searching for regions with GFP expression in *PdfR(B)GAL4* and the absence of expression in these regions in *PdfR(A)GAL4*. Strong GFP immunoreactivity was observed in neuronal cell bodies located near the sub-esophageal ganglion (SOG), in the thoracic ganglion and the antennal mechanosensory and motor complex (AMMC) (Figure S4D) in *PdfR(B)GAL4* (Figure 8A, 8C, 8E, 8G and 8H). Expression in these regions was reduced in *PdfR(A)GAL4* (Figure 8B, 8D, 8F, 8I and 8J). Expression was also seen in other regions of the brain including the medial neurosecretary cells (mNSCs), where *PdfR(B)GAL4* and *PdfR(A)GAL4* expressed to equivalent levels (Figure 8K and 8L). A summary of the complete expression patterns of both the GAL4 lines is shown in Figure 8M. The expression analysis suggests that PdfR function in neurons of the AMMC, SOG and thoracic ganglion regulates the maintenance of flight in *Drosophila*.

## Discussion

**Figure 9:**
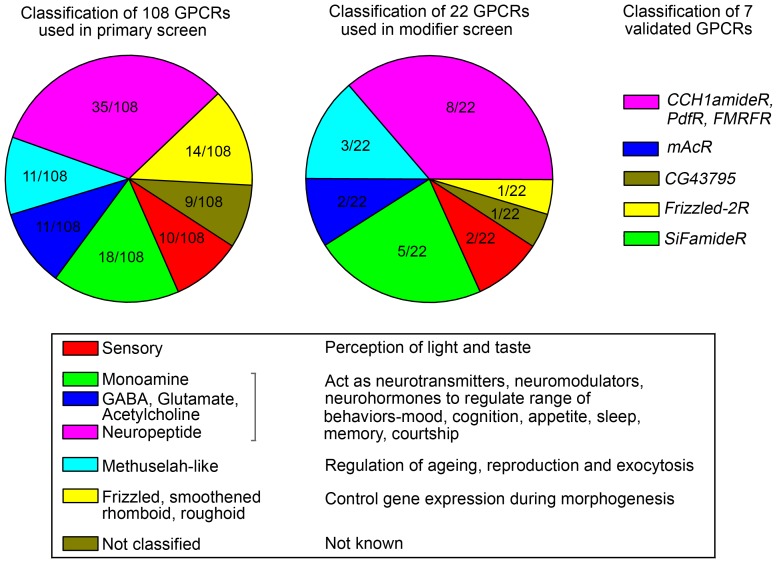
A summary of GPCRs that regulate flight in *Drosophila*. Classification of GPCRs that were used for the primary screen, the secondary suppressor screen and finally validated as activators of IP_3_/Ca^2+^ signaling in the context of *Drosophila* flight.

In a genetic RNAi screen for GPCRs that regulate flight, twenty-two genes were identified amongst which eight encoded neuropeptide receptors and seven were for neurotransmitter receptors, highlighting the importance of these ligands for neuro-modulation of motor function (Figure 9). The remaining genes encoded various receptor classes with possible roles in development like the frizzled-2 receptor and the methuselah-like receptors (3/22), putative sensory receptors (rhodopsin-like and trehalose-sensing) and *CG43795* with no clear homology to any class of GPCRs. Despite testing multiple RNAi lines for each receptor, our screening strategy may have missed out some flight regulating GPCRs. This would be true specifically in cases where RNAi lines tested for a particular gene were not efficacious, if the pan-neuronal GAL4 strain utilized in the screen expressed weakly in the cognate neurons and due to inappropriate temporal expression of the GAL4 with respect to the temporal requirement for that receptor.

In a secondary modifier screen designed to test if the signaling mechanism activated by the identified receptors was indeed intracellular Ca^2+^ release and store-operated Ca^2+^ entry, flight deficits in three out of the twenty two receptors identified (*CG34411*, *CG3171* and *CG43795*) were further enhanced by expression of either *AcGq* or *dSTIM* ^+^ suggesting that these receptors could constitute an inhibitory signaling component of the flight circuit. Inhibitory neural circuits within central pattern generators constitute an integral part of any rhythmic motor behavior [2]. When analyzed by us, the predicted protein sequence of *CG43795* exhibits highest homology with the predicted sequence of *CG31760* (E = 1.262e-66), which in turn is classified as a putative glutamate/GABA receptor. Similar to vertebrates, GABA functions as an inhibitory neurotransmitter in *Drosophila* [32]. The role of *CG43795*, *CG34411* and *CG3171* as putative components of inhibitory signaling during acute flight in adults requires further study.

Amongst the five receptors identified in the secondary suppressor screen, two have been linked with IP_3_-mediated Ca^2+^ release previously. The mAcR can stimulate the IP_3_R in transfected S2 cells [17], [18], [33], [34] and by over-expression in primary neuronal cultures [4]. Similarly, the FmrfR was shown to modulate intracellular Ca^2+^ in type 1 nerve terminals and thus regulate light-dependant escape behavior in *Drosophila* larvae [35]. However, a physiological role for these receptors in the regulation of flight in adult *Drosophila* has not been described earlier. The temporal analysis showed a dual requirement for the FmrfR during pupal stages and in adults, which were non-additive, suggesting that the same set of neurons require FmrfR function during development and for modulating acute flight in adults. The precise neurons that require FmrfR function for maintenance of flight and the role of IP_3_/Ca^2+^ signaling in them, needs further analysis.

Another recently de-orphanised receptor identified in the final screen was CCH1aR with the specific ligand, CCH1amide [36]. The ligand is found in the *Drosophila* mid-gut and central nervous system [37]. However, physiological functions have not been attributed to the CCH1aR so far. From the differential effect on flight obtained by knockdown of the CCH1aR, mAcR, FmrfR and PdfR as well as their differential temporal requirement, it seems likely that each receptor regulates independent aspects of either flight circuit development, function or both. This hypothesis needs further confirmation by genetic and anatomical studies for each receptor.

Neuroanatomical studies for spatial localization of the identified receptors in the context of flight circuit components are required, as has been attempted here for the PdfR. From previous work we know that synaptic function of the well-characterized Giant Fibre Pathway, required for the escape response, is normal in IP_3_R mutants [3], [38], [39]. Instead, intracellular calcium signaling is required for development of the air-puff stimulated flight circuit, which is a laboratory paradigm of voluntary flight. Spontaneous calcium transients through voltage gated Ca^2+^ channels can affect dendritic morphology and neurotransmitter specification in developing neural circuits [40], [41]. The developmental processes that require intracellular calcium signaling during flight circuit maturation may be similar but are not understood so far. In part, a reason for this lack of understanding is the absence of well characterized interneurons that integrate and communicate sensory information to the flight motor pathways in the context of voluntary flight. Thus, spatial localization of GPCRs found in this screen and neural connections of flight GPCR expressing cells will in future help understand and identify both neural components of the voluntary flight circuit and the role of intracellular calcium signaling in flight circuit maturation and function.

The screen also identified the SiFaR as a neuronal receptor required for viability. However, since pupal lethality in *SiFaR* knockdown was not suppressed by expression of *AcGq*, the downstream signaling mechanism of this receptor remains unclear. A recent study in the Blacklegged Tick has implicated SiFaR in the regulation of feeding [42]. It is therefore possible that, lethality in *SiFaR* knockdown animals is a consequence of reduced feeding at the larval stages.

Analysis of flight phenotypes exhibited by knockdown of *dFz-2R* suggests a requirement for this receptor during flight circuit development. Suppression of flight deficits in *dsFz-2R* expressing flies by over expression of *dSTIM* ^+^, but not *AcGq* indicates that this receptor does not activate the canonical GPCR/IP_3_/Ca^2+^ signaling mechanism. From previous studies, it is known that dFz-2R can signal through Wnt/βcatenin pathway [43], [44] while recent speculations implicate a non-canonical Wnt-Ca^2+^ pathway as downstream of Fz-2R [45], [46] already shown for rat (*rFz-2R*) and Xenopus (*XFz-2R*) [47]. In *Drosophila*, dFz-2R is thought to act via the G-protein Gαo [48]. Suppression of *dsFz-2R* flight deficits by *dSTIM^+^* implicates intracellular Ca^2+^ signaling as downstream of dFz-2R activation for the first time in *Drosophila*. While the cellular correlates of dFz-2R activation need to be demonstrated directly in *Drosophila* flight circuit neurons, it is likely that this study will help identify other molecular components of this pathway.

Interestingly, we discovered a regulatory role for the PdfR in *Drosophila* flight where our genetic data implicate IP_3_-mediated Ca^2+^ release as the downstream signaling mechanism. Although, PdfR stimulation increases cAMP levels in HEK293 cells transfected with *Drosophila PdfR*, it is also known that cellular Ca^2+^ levels increase moderately in response to PDF [22]. Our findings suggest that the PdfR is capable of stimulating dual G-proteins, similar to 5HT-dro2A and 5HT-dro2B, the cellular responses of which include a decrease in cAMP as well as an increase in inositol phosphates in response to serotonin [49], [50]. The role of the neuropeptide ligand, PDF and PdfR in regulation of circadian rhythms is well documented in adults [22], [51], [52] and more recently during early larval development for instructing circadian circuit formation in pupae [53]. In addition PDF function in circadian neurons regulates several other processes like reproduction, arousal and geotaxis [22], [54], [55]. Recently, the PdfR orthologue in *C.elegans* was found to modulate locomotory behavior [56]. Our data support earlier published data in *Drosophila* suggesting that PdfR can be activated by ligands other than PDF, such as vertebrate PACAP (pituitary adenylate cyclase activating polypeptide) [22]. In vertebrates, signaling by PACAP modulates locomotor activity and the exploratory behavior of rats, mice, chicken and goldfish [57]. Our study shows that *PdfR(B)GAL4* expressing neurons which rescue circadian rhythm phenotypes of *PdfR* mutants (*PdfR* ***^3369^***, *PdfR* ***^5304^***; [30]) also function in flight regulation. The source of PDF and/or another ligand that activates PdfR signaling in the context of flight remains to be determined. PDF is secreted from two known sources in *Drosophila*; one is the lateral ventral protocerebrum (LNvs) and the other is neurons in the abdominal ganglion (AbNs; [58]–[60]). A recent study revealed an endocrine mode of action of PDF for the regulation of ureter contractions [61]. A better understanding of the neurocircuitry underlying voluntary flight is required to distinguish between these two sources of PDF for development and function of the flight circuit, as well as to investigate whether endocrine mechanisms deliver the ligand(s) for activating the PdfR in the context of flight. This study adds to the growing body of evidence which suggests that signaling through PdfR could serve as a global integrator of a repertoire of behaviors important for *Drosophila* survival in the wild.

## Materials and Methods

### Fly rearing and stocks

*Drosophila* strains were reared on corn flour/agar media supplemented with yeast, grown at 25°C, unless otherwise mentioned in the experimental design. The wild-type *Drosophila* strain used was *Canton-S (CS)*. The pan-neuronal GAL4 driver used was *Elav^C155^GAL4* obtained from Bloomington Stock Center, Bloomington, IN. *UAS-PdfR16L*, *Pacman PdfR-myc 70*, *PdfR(B)GAL4(2)* and *PdfR(A)GAL4(2)* were obtained from Paul Taghert (Washington University, St. Louis) [30]. G-protein coupled receptor *UASRNAi* lines were obtained from Vienna *Drosophila* RNAi center, Vienna, Austria (VDRC) and National Institute of Genetics Fly Stocks Centre, Kyoto, Japan (NIG). The *UAS-RNAi* strains for *dSTIM* (*dsdSTIM*, 47073) and *dOrai* (*dsdOrai*, 12221) were obtained from VDRC and for *itpr* (*dsitpr*, 1063-R2) from NIG [4]. The other strains used were as follows: *UASdSTIM^+^* [24], *UASAcGq3* [27], *GAL80^ts^* with two inserts on second chromosome (generated by Albert Chiang, NCBS, Bangalore, India). *UASdicer(X)*, used in combination with *dsdSTIM* and *dsdOrai*; *UASdicer(III)*, used in combination with *dsitpr* and *UASmCD8GFP(II)* were obtained from BDSC. The other fly strains used were generated using standard *Drosophila* genetic methods.

### Flight assay video and electrophysiological recordings

Females of the *Elav^C155^GAL4* strain were mated with males of each RNAi strain. In the resulting progeny, male flies gave varied responses (data not shown) to air-puff induced flight. Therefore only adult female flies were used further for analysis. Adult females were collected soon after eclosion and aged for 3–4 days before testing for flight. Flies were anaesthetized on ice for 15 min and a thin metal wire was glued between the neck and thorax region with the help of nail polish. To test for air-puff responses, videos were recorded for 30 sec after giving a gentle mouth-blown air puff stimulus to the tethered fly. These videos were analyzed and percentage flight time was calculated. For each RNAi line, 10 flies were tethered and tested along with 10 control flies. Physiological recordings were obtained from the indirect Dorsal Longitudinal Muscles (DLMs) as described previously [3]. Briefly, an un-insulated 0.127 mm tungsten electrode, sharpened by electrolysis to attain 0.5 µm tip diameter, was inserted in the DLM (fiber a). A similar electrode was inserted in the abdomen for reference. Spontaneous firing was recorded for 2 min and air-puff stimulated recordings were done for 30 s. All recordings were done using an ISO-DAM8A amplifier (World Precision Instruments, Sarasota, FL) with filter set up of 30 Hz (low pass) to 10 kHz (high pass). Gap free mode of pClamp8 (Molecular Devices, Union City, CA) was used to digitize the data (10 kHz) on a Pentium 5 computer equipped with Digidata 1322A (Molecular Devices). Data were analyzed using Clampfit (Molecular Devices) and the mean and standard error (SEM) were plotted using Origin 7.5 software (MicroCal, Origin Lab, Northampton, MA, USA).

### RNA isolation and qPCR

For isolation of RNA, the central nervous system (CNS) was dissected from 3^rd^ instar wandering larvae. Each sample of RNA was extracted from five CNSs and three independent preparations were analyzed for each experiment. Total RNA was isolated using TRIzol Reagent (Invitrogen Life Technologies, Carlsbad, CA, USA) according to the manufacturer's specifications. Integrity of RNA was confirmed by visualization on a 1% TAE (40 mM Tris pH 8.2, 40 mM acetate, 1 mM EDTA) agarose gel. Total RNA (500 ng) was treated with DNase in a volume of 45.5 µl with 1 µl (1 U) DNase I (Amplification grade, Invitrogen Life Technologies, Carlsbad, CA, USA) with 1 mM dithiothreitol (DTT) (Invitrogen Life Technologies, Carlsbad, CA, USA), 40 U of RNase Inhibitor (Promega, Madison, WI, USA) in 5× First Strand Buffer (Invitrogen Life Technologies, Carlsbad, CA, USA) for 30 min at 37°C and heat inactivated for 10 min at 70°C. The reverse transcription reaction was performed in a final volume of 50 µl by addition of 1 µl (200 U) Moloney murine leukemia virus (M-MLV) reverse transcriptase (Invitrogen Life Technologies, Carlsbad, CA, USA), 2.5 µl (500 ng) random hexaprimers (MBI Fermentas, Glen Burnie, MD, USA) and 1 µl of a 25 mM dNTP mix (GE Healthcare, Buckinghamshire, UK). Samples were incubated for 10 min at 25°C, then 60 min at 42°C and heat inactivated for 10 min at 70°C. The polymerase chain reactions (PCRs) were performed using 1 µl of cDNA as a template in a 25 µl reaction under appropriate conditions. Real time quantitative PCR (qPCR) were performed on an ABI 7500 Fast machine (Applied Biosystems, Foster City, California, USA) operated with ABI 7500 software version 2 (Applied Biosystems, Foster City, California, USA) using MESA GREEN qPCR MasterMIx Plus for SYBR Assay I dTTp (Eurogentec, Belgium). qPCRs were performed with *rp49* primers as internal controls and primers specific to gene of interest using dilutions of 1∶10. Sequences of the primers used in the 5′ to 3′ directions are given below. The sequence of the forward primer is given first in each case:

*rp49* CGGATCGATATGCTAAGCTGT; GCGCTTGTTCGATCCGTA,

*Fz-2R* GGTTACGGAGTGCCAGTCAT; CACAGGAAGAACTTGAGGTCC,

*mAcR* CAAGGACGAGTGCTACATCC; CCTAAATCAGAAGGCTCCTCC,

*CCH1aR* GACCAAAGGAATGGCGTAGTAG; CGCTCGCATCCACAGTTTAC,

*PdfR* CAAATGCCACGGAGGTGAATC; TCAGCAGGGAAACTATAAGGGC,

*FmrfR* GTGCGAAAGTTACCCGTCG; TAATCGTAGTCCGTGGGCG,

*SiFaR* CAATCAGTGTGGCTGGCAG; CCTACATCGTCGTCTTCCTG.

Each qPCR experiment was repeated three times with independently isolated RNA samples. The cycling parameters were 95°C for 5 min, followed by 40 cycles of 95°C for 15 s and 60°C for 1 min. The fluorescent signal produced from the amplicon was acquired at the end of each polymerization step at 60°C. A melt curve was performed after the assay to check for specificity of the reaction. The fold change of gene expression in the mutant relative to wild-type was determined by the comparative ΔΔCt method [62]. In this method the fold change = 2^−ΔΔCt^ where ΔΔCt = (C_t(target gene)_−C_t(rp49)_)_mutant2_−(C_t(target gene)_−C_t(rp49)_)_Wild type_.

### Immunohistochemistry

Immunohistochemistry was performed on *Drosophila* adult brains expressing cytosolic GFP (*UASGFP*) with the specified *GAL4* strains, after fixing the dissected tissue in 4% paraformaldehyde. The following primary antibodies were used: mouse monoclonal nc82 antibody (1∶20, kindly provided by Eric Buchner), rabbit anti-GFP antibody (1∶10,000; #A6455, Molecular Probes, Eugene, OR, USA). Fluorescent secondary antibodies were used at a dilution of 1∶400 as follows: anti-rabbit Alexa Fluor 488 (#A1108) and anti-mouse Alexa Fluor 568 (#A1104, Molecular Probes, Eugene, OR, USA). Confocal analysis was performed on an Olympus Confocal FV1000 microscope. Confocal data were acquired as image stacks of separate channels and combined and visualized as three-dimensional projections using the FV10-ASW 1.3 viewer (Olympus Corporation, Tokyo, Japan).

### Western blots

Adult brains and thoracic ganglia were dissected from 3 to 5 day old progeny of the indicated genotypes. Protein extracts were made by homogenizing the sample in homogenizing buffer (40 mM Tris pH 7.4, 1 mM EDTA, 1 mM EGTA, 0.05% Triton X-100) and were separated on a 6% SDS-polyacrylamide gel and transferred to nitrocellulose membrane by standard western blotting protocols. The affinity purified anti-InsP_3_R rabbit polyclonal antibody (IB-9075; [34]) was used at a dilution of 1∶300. A mouse anti-spectrin antibody (3A9) (1∶50 dilution, Developmental Studies Hybridoma Bank, University of Iowa, Iowa) was used as a loading control for the InsP_3_R. Two anti-dSTIM mouse antibodies (8G1) and (3C1) mixed 1∶1 (Generated by Bioneeds, Bangalore, India) were used at a dilution of 1∶200. The mouse anti-GFP monoclonal antibody (sc-9996, Santa Cruz Biotechnology, CA) was used at a dilution of 1∶1000. The mouse anti-β-tubulin monoclonal antibody (E7, Developmental Studies Hybridoma Bank, University of Iowa, Iowa) was used at a dilution of 1∶200 as a loading control for dSTIM and GFP. Secondary antibodies conjugated to horseradish peroxidase were used, and protein was detected in the blot by addition of a chemiluminescent substrate from Thermo Scientific (No. 34075; Rockford, IL, USA).

## Supporting Information

Figure S1 G-protein coupled receptors that regulate wing expansion. Pan-neuronal knockdown of the 5HT1a receptor (*16720-2*), neuropeptide F receptor (*1147-2*), dromyosuppressin receptor 1 *(8985-4*) and methuselah- like 7 receptor (*7476-3*) resulted in wing posture and wing expansion defects in adults. Percentages of flies exhibiting the indicated phenotype are shown for males and females of each genotype.
(TIF)

Figure S2 Genetic validation of GPCRs as IP_3_/Ca^2+^ linked by pan-neuronal expression of *AcGq* and *dSTIM^+^*. The grey bars for each RNAi strain represent percentage flight time of adults with pan-neuronal knockdown of the indicated GPCR. The blue and green bars represent the percentage flight time by additional expression of either *AcGq* (green) or *dSTIM^+^* (blue). Pan-neuronal *GAL4* controls (grey), with *AcGq* (green) and *dSTIM^+^* (blue) showed normal flight. Open circles within the bars represent percentage flight times for individual flies. Where multiple animals gave the same flight time, the circles are overlapping. Percentage flight time was obtained by measuring flight in single flight assays from 20 flies of each genotype. (**P<0.01, *P<0.05, obtained by one way ANOVA tests, where the rescues were compared to pan-neuronal knockdown of the respective GPCR).
(TIF)

Figure S3 Quantification of GPCR gene transcripts in larval brains after pan-neuronal expression of GPCR specific RNAi. The Ct values for each gene (indicated by individual CG numbers) were normalized to the level of a housekeeping gene (*rp49*) in control RNA from CS larvae of an equivalent developmental stage. The Y-axis represents log2 fold changes calculated by the ΔΔCt method. Each value is the mean ± SEM of three independent experiments, obtained from three independent RNA samples. RNA was extracted from larval brains expressing a GPCR RNAi that gave a flight deficit and from an RNAi strain for the same GPCR, in which the flight deficit was not observed. Gene expression was significantly reduced for the RNAi strains that gave a flight defect when compared to the expression of that gene in the pan-neuronal GAL4 control (*P<0.05, **P<0.005; Student's *t* test). Expression level of a representative GPCR, as described in materials and methods, is shown in the first bar. Normal levels of gene expression were observed in the RNAi strains that did not give any flight defect.
(TIF)

Figure S4 Expression pattern of *PdfR(B)GAL4* and *PdfR(A)GAL4* in larval brain, adult brain and thoracic ganglion. Expression of PdfR using *PdfR(B)GAL4* (A) and *PdfR(A)GAL4* (B) (green:antiGFP) in 3^rd^ instar larval brain. C) Level of expression of GFP in protein extracts of adult brains plus thoracic ganglia was assessed by western blots. The strength of expression of both GAL4 strains was similar. D) Schematic of adult brain showing regions of interest: mNSC: medial neurosecretory cells, VLP: ventrolateral protocerebrum, AMMC: antennal mechanosensory and motor complex, SOG: subesophageal ganglion, T1: thoracic region 1, T2: thoracic region 2, T3: thoracic region 3, Ab: abdominal region. E, F) Expression of PdfR expressing neurons (green: anti-GFP) in adult brain with *PdfR(B)GAL4* and *PdfR(A)GAL4*. Neuropils of the brain are shown in magenta by anti-NC82 staining.
(TIF)

Movie S1 Real time video recording of air-puff induced flight in the following genotypes from left to right. 1) *ElavC155GAL4; GAL80^ts^*, 2) *ElavC155GAL4; GAL80^ts^; dsFz-2R* and 3) *dsFz-2R/+*. All flies were grown at 29°C during pupal stages and were prepared for recording as described in materials and methods. Following a gentle air-puff *ElavC155GAL4;GAL80^ts^;dsFz-2R* animals could initiate flight but were not able to sustain it for as long as control flies of the genotypes *ElavC155GAL4;GAL80^ts^* and *dsFz-2R/+*.
(AVI)

Movie S2 Real time video recording of air-puff induced flight in, from left to right, *ElavC155GAL4;GAL80^ts^*, *ElavC155GAL4;GAL80^ts^;dsFmrfR* and *dsFmrfR/+*. RNAi was induced only in adults (29°C shift post-eclosion). Flies were prepared for recording as described in materials and methods. Following a gentle air-puff *ElavC155GAL4;GAL80^ts^;dsFmrfR* could initiate flight but were unable to sustain flight as long as control flies of the genotypes *ElavC155GAL4;GAL80^ts^* and *dsFmrfR/+*.
(AVI)

Table S1 List of GPCRs, and their respective RNAi lines tested in the primary screen, are shown, along with the percentage flight time observed upon pan-neuronal expression of each RNAi line.
(DOC)
