# Supplementary figures and images for: A Genetic RNAi Screen for IP3/Ca2+ Coupled GPCRs in Drosophila Identifies the PdfR as a Regulator of Insect Flight
Source: PLoS Genet. 2013 Oct 3;9(10):e1003849. doi: 10.1371/journal.pgen.1003849 (PMC3789835; doi:10.1371/journal.pgen.1003849)

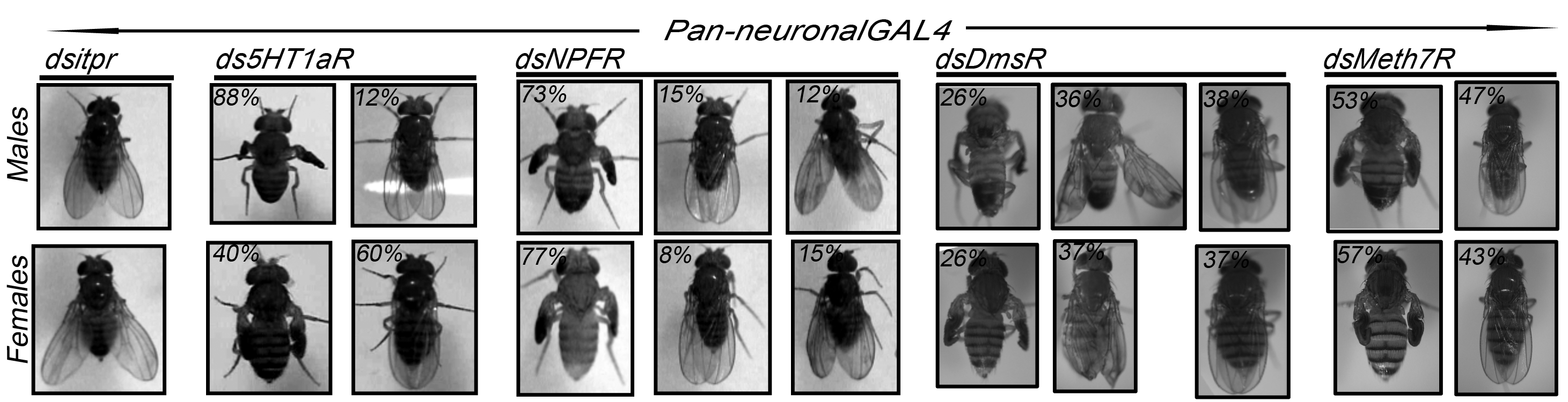

Supplement: Figure S1 — G-protein coupled receptors that regulate wing expansion. Pan-neuronal knockdown of the 5HT1a receptor (16720-2), neuropeptide F receptor (1147-2), dromyosuppressin receptor 1 (8985-4) and methuselah- like 7 receptor (7476-3) resulted in wing posture and wing expansion defects in adults. Percentages of flies exhibiting the indicated phenotype are shown for males and females of each genotype. (TIF) [file pgen.1003849.s001.tif]

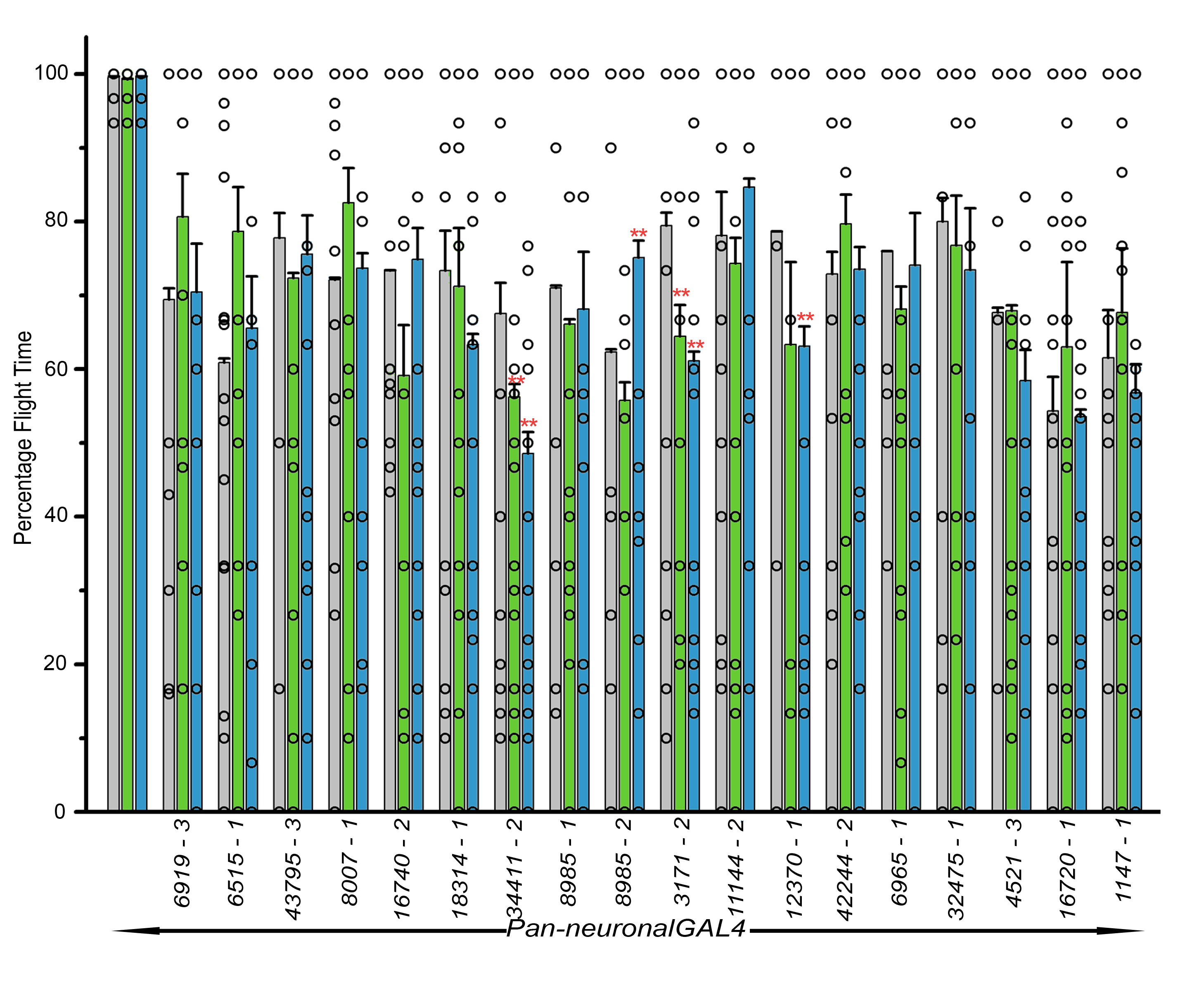

Supplement: Figure S2 — Genetic validation of GPCRs as IP3/Ca2+ linked by pan-neuronal expression of AcGq and dSTIM+. The grey bars for each RNAi strain represent percentage flight time of adults with pan-neuronal knockdown of the indicated GPCR. The blue and green bars represent the percentage flight time by additional expression of either AcGq (green) or dSTIM+ (blue). Pan-neuronal GAL4 controls (grey), with AcGq (green) and dSTIM+ (blue) showed normal flight. Open circles within the bars represent percentage flight times for individual flies. Where multiple animals gave the same flight time, the circles are overlapping. Percentage flight time was obtained by measuring flight in single flight assays from 20 flies of each genotype. (**P<0.01, *P<0.05, obtained by one way ANOVA tests, where the rescues were compared to pan-neuronal knockdown of the respective GPCR). (TIF) [file pgen.1003849.s002.tif]

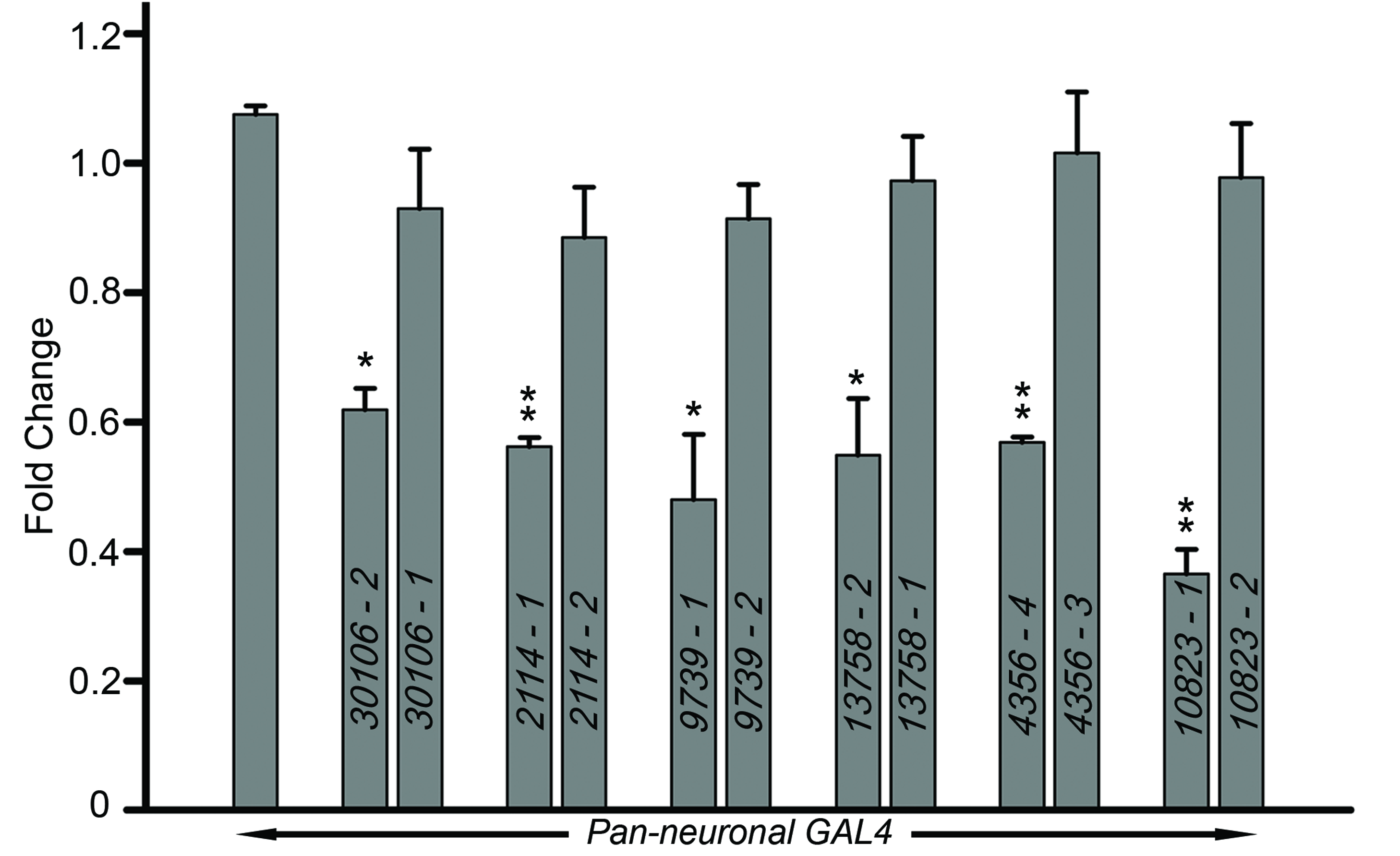

Supplement: Figure S3 — Quantification of GPCR gene transcripts in larval brains after pan-neuronal expression of GPCR specific RNAi. The Ct values for each gene (indicated by individual CG numbers) were normalized to the level of a housekeeping gene (rp49) in control RNA from CS larvae of an equivalent developmental stage. The Y-axis represents log2 fold changes calculated by the ΔΔCt method. Each value is the mean ± SEM of three independent experiments, obtained from three independent RNA samples. RNA was extracted from larval brains expressing a GPCR RNAi that gave a flight deficit and from an RNAi strain for the same GPCR, in which the flight deficit was not observed. Gene expression was significantly reduced for the RNAi strains that gave a flight defect when compared to the expression of that gene in the pan-neuronal GAL4 control (*P<0.05, **P<0.005; Student's t test). Expression level of a representative GPCR, as described in materials and methods, is shown in the first bar. Normal levels of gene expression were observed in the RNAi strains that did not give any flight defect. (TIF) [file pgen.1003849.s003.tif]

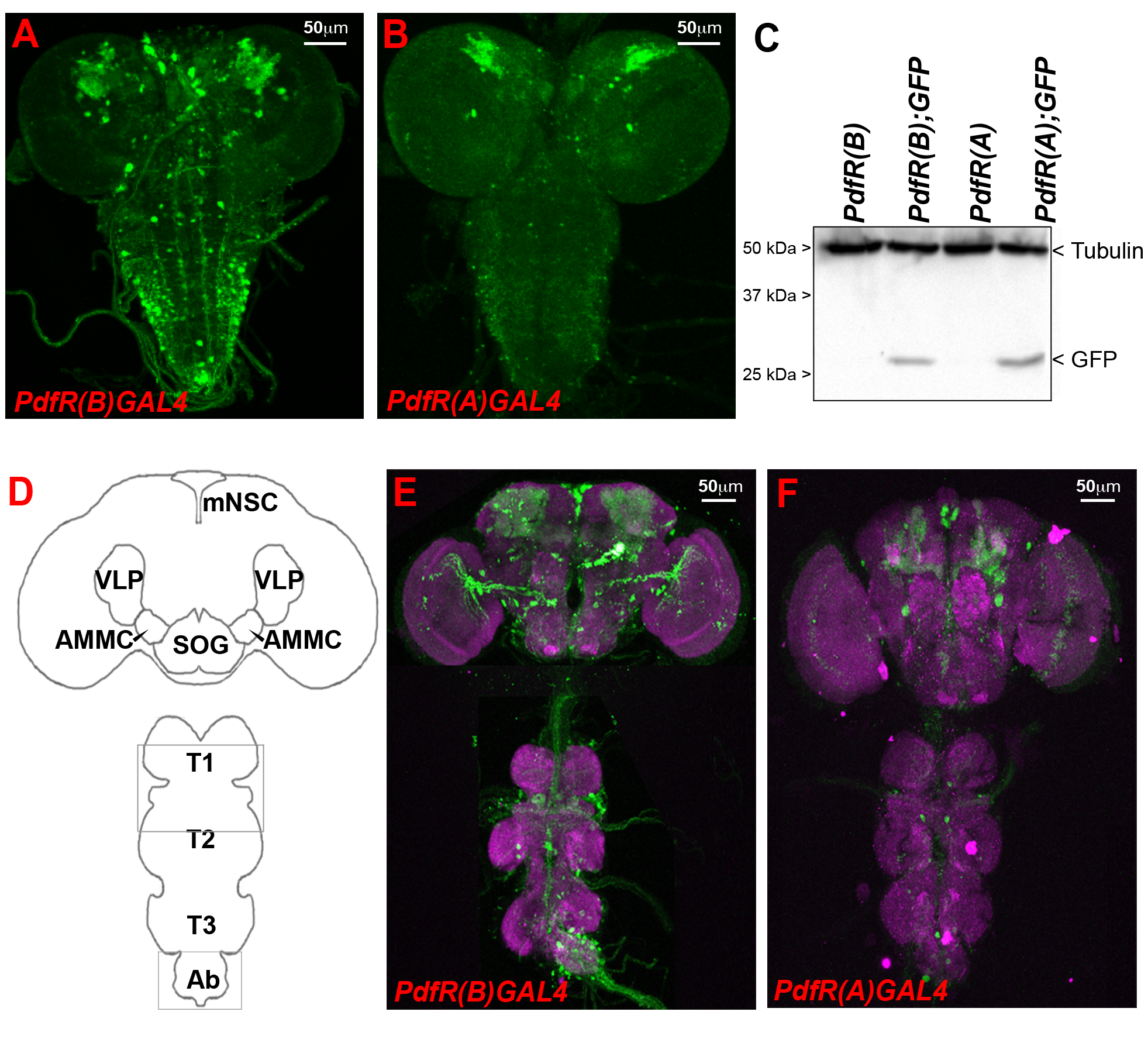

Supplement: Figure S4 — Expression pattern of PdfR(B)GAL4 and PdfR(A)GAL4 in larval brain, adult brain and thoracic ganglion. Expression of PdfR using PdfR(B)GAL4 (A) and PdfR(A)GAL4 (B) (green:antiGFP) in 3rd instar larval brain. C) Level of expression of GFP in protein extracts of adult brains plus thoracic ganglia was assessed by western blots. The strength of expression of both GAL4 strains was similar. D) Schematic of adult brain showing regions of interest: mNSC: medial neurosecretory cells, VLP: ventrolateral protocerebrum, AMMC: antennal mechanosensory and motor complex, SOG: subesophageal ganglion, T1: thoracic region 1, T2: thoracic region 2, T3: thoracic region 3, Ab: abdominal region. E, F) Expression of PdfR expressing neurons (green: anti-GFP) in adult brain with PdfR(B)GAL4 and PdfR(A)GAL4. Neuropils of the brain are shown in magenta by anti-NC82 staining. (TIF) [file pgen.1003849.s004.tif]
