# Supplementary material for: A Genetic RNAi Screen for IP3/Ca2+ Coupled GPCRs in Drosophila Identifies the PdfR as a Regulator of Insect Flight
Source: PLoS Genet. 2013 Oct 3;9(10):e1003849. doi: 10.1371/journal.pgen.1003849 (PMC3789835; doi:10.1371/journal.pgen.1003849)
Supplement: Table S1 — List of GPCRs, and their respective RNAi lines tested in the primary screen, are shown, along with the percentage flight time observed upon pan-neuronal expression of each RNAi line. (DOC) [file pgen.1003849.s007.doc]

Table S1: List of GPCRs and their respective RNAi line tested in the primary screen are shown, with the percentage flight time observed upon pan-neuronal expression of these RNAi lines.

| **CG (Gene Name)** | **RNAi Line (NIG/VDRC)⌃** | **Name used in text** | **Phenotype (pan-neuronal)** | **RNAi hetero-zygotes** |
| --- | --- | --- | --- | --- |
| CG4322 (moody) | 4322R-1 III | 4322 - 1 | 100 | 100 |
| CG4550 (ninaE) | 4550R-1 II | 4550 - 1 | 93 | 100 |
|  | 4550R-2 III | 4550 - 2 | 96.66 | 94.33 |
| CG16740 (Rh2) | 16740R-2 III | 16740 - 1 | 99.33 | 94 |
|  | 16740R-4 III | 16740 - 2 | 73.33 | 99.66 |
| CG10888 (Rh3) | 10888R-1 II | 10888 - 1 | 89.66 | 96 |
|  | 10888R-2 II | 10888 - 2 | 97.66 | 99 |
| CG9668 (Rh4) | 9668R-1 III | 9668 - 1 | 99.33 | 100 |
|  | 9668R-2 III | 9668 - 2 | 96.66 | 100 |
| CG5279 (Rh5) | NONE | NA | NA | NA |
| CG5192 (Rh6) | NONE | NA | NA | NA |
| CG5638 (Rh7) | NONE | NA | NA | NA |
| CG12073 (5-HT7) | 104804 | 12073 - 1 | 94.66 | 99 |
| CG1056 (5-HT2) | 102105 | 1056 - 1 | 100 | 100 |
| CG8007 | 11461 | 8007 - 1 | 72.66 | 100 |
|  | 11462 | 8007 - 2 | 96.33 | 100 |
|  | 102356 | 8007 - 3 | 94 | 100 |
| CG16720 (5-HT1A) | 16720 R -1(III) | 16720 - 1 | 66.33 | 100 |
| CG15113 (5-HT1B) | 9559 | 15113 - 1 | 90 | 99.66 |
|  | 46485 | 15113 - 2 | 96.66 | 100 |
| CG9652 (DopR) | 107058 | 9652 - 1 | 86 | 98 |
| CG33517 (D2R) | 17004R-2 II | 33517 - 1 | 97 | 100 |
|  | 17004R-3 II | 33517 - 2 | 100 | 100 |
|  | 11470 | 33517 - 3 | 97 | 100 |
|  | 11471 | 33517 - 4 | 94.33 | 100 |
| CG4356 (mAcR-60C) | 4356R-1 III | 4356 - 1 | 81.66 | 100 |
|  | 4356R-3 II | 4356 - 2 | 91.33 | 100 |
|  | 33123 | 4356 - 3 | 100 | 100 |
|  | 101407 | 4356 - 4 | 58 | 99.66 |
| CG7918 | 7918R-1 II | 7918 - 1 | 100 | 100 |
|  | 7918R-3 II | 7918 - 2 | 93.33 | 99.66 |
|  | 100217 | 7918 - 3 | 100 | 100 |
| CG14575 (capaR) | 105556 | 14575 - 1 | 93.33 | 100 |
| CG7485 (Oct-TyrR) | 26876 | 7485 -1 | 100 | 100 |
|  | 26877 | 7485 - 2 | 83.33 | 99 |
| CG2114 (Fmrf R) | 2114R-1 III | 2114 - 1 | 77.66 | 100 |
|  | 2114R-2 III | 2114 - 2 | 100 | 100 |
|  | 9594 | 2114 - 3 | 97.66 | 100 |
| CG8985 (DmsR-1) | 8985R-2 II | 8985 - 1 | 78 | 96.66 |
|  | 8985 R-3 III | 8985 - 2 | 62 | 99 |
|  | 101845 | 8985 - 3 | 89.33 | 100 |
| CG13803 (DmsR-2) | 13803R-1 III | 13803 - 1 | 100 | 100 |
|  | 13803R-3 II | 13803 - 2 | 99.33 | 100 |
| CG33639 | 29644 | 33369 - 1 | 96 | 100 |
|  | 108753 | 33639 - 2 | 100 | 100 |
| CG13229 | 13229R-1 III | 13229 - 1 | 95 | 100 |
|  | 13229R-2 II | 13229 - 2 | 96 | 100 |
|  | 100433 | 13229 - 3 | 53.33 | 100 |
| CG3171 (Tre1) | 3171R-2 III | 3171 - 1 | 99.33 | 100 |
|  | 3171R-3 II | 3171 - 2 | 75.33 | 100 |
| CG4313 | 107434 | 4313 - 1 | 83.33 | 99 |
| CG12290 | 12290R-1 II | 12290 - 1 | 93.33 | 100 |
|  | 12290R-3 | 12290 - 2 | 88.33 | 99 |
|  | 100939 | 12290 - 3 | 95.33 | 100 |
|  | 1246 | 12290 - 4 | 94.66 | 100 |
|  | 1247 | 12290 - 5 | 99.66 | 100 |
| CG6986 (Proc-R) | 7217 | 6986 - 1 | 100 | 100 |
| CG13579 | 13579R-1 (III) | 13579 - 1 | 98 | 99 |
|  | 13579R-2 (X) | 13579 - 2 | 100 | 100 |
|  | 110022 | 13579 - 3 | 99.33 | 100 |
|  | 9366 | 13579 - 4 | 99.33 | 100 |
| CG13995 | 13995R-1 III | 13995 - 1 | 100 | 100 |
|  | 13995R-3 III | 13995 - 2 | 95 | 100 |
|  | 102804 | 13995 - 3 | 97.3 | 100 |
|  | 42524 | 13995 - 4 | 100 | 100 |
|  | 42525 | 13995 - 5 | 100 | 100 |
| CG7497 | 106421 | 7497 - 1 | 98.66 | 100 |
| CG4395 (hec) | 7223 | 4395 - 1 | 91.66 | 100 |
| CG32843 (Dh31-R1) | 17043R-1 II | 32843 - 1 | 91.66 | 100 |
|  | 8777 | 32843 - 2 | 91 | 100 |
|  | 101995 | 32843 - 3 | 96.33 | 100 |
| CG13758 (Pdfr) | 42724 | 13758 - 1 | 92.66 | 100 |
|  | 106381 | 13758 - 2 | 46.66 | 100 |
| CG8422 (Dh44-R1) | NONE | NA | NA | NA |
| CG12370 (Dh44-R2 | 12370R-2 II | 12370 - 1 | 78.66 | 100 |
|  | 12370R-4 III | 12370 - 2 | 93.33 | 100 |
|  | 102292 | 12370 - 3 | 88.33 | 100 |
|  | 109558 | 12370 - 4 | 94.33 | 100 |
| CG11318 | 108136 | 11318 - 1 | 92.66 | 100 |
| CG15556 | 15556R-1 III | 15556 - 1 | 80 | 100 |
|  | 101118 | 15556 - 2 | 100 | 100 |
| CG8639 | 29968 | 8639 - 1 | 97.66 | 100 |
|  | 29969 | 8639 - 2 | 95.66 | 100 |
|  | 100749 | 8639 - 3 | 100 | 100 |
| CG6936 (mth) | NONE | NA | NA | NA |
| CG4521 (mthl1) | 4521R-1 X | 4521 - 1 | 94.33 | 100 |
|  | 4521R-2 II | 4521 - 2 | 96.66 | 100 |
|  | 107488 | 4521 - 3 | 68.33 | 100 |
| CG17795 (mthl2) | 26815 | 17795 - 1 | 94 | 100 |
| CG6530 (mthl3) | 49623 | 6530 - 1 | 96 | 100 |
|  | 49624 | 6530 - 2 | 88.66 | 100 |
|  | 104033 | 6530 - 3 | 86.66 | 100 |
| CG6536 (mthl4) | NONE | NA | NA | NA |
| CG6965 (mthl5) | 3390 | 6965 - 1 | 76 | 99 |
|  | 101593 | 6965 – 2 | 90 | 100 |
| CG7476 (mthl7) | 46332 | 7476 - 1 | 100 | 100 |
|  | 46333 | 7476 - 2 | 82.66 | 100 |
| CG32475 (mthl8) | 4071 | 32475 - 1 | 65.33 | 96.66 |
| CG17084 (mthl9) | 17084 R-1 II | 17084 - 1 | 91.66 | 99 |
|  | 17084 R-3 II | 17084 - 2 | 92.66 | 100 |
|  | 108967 | 17084 - 3 | 98 | 100 |
| CG17061 (mthul10) | 51425 | 17061 - 1 | 98 | 100 |
|  | 51426 | 17061 - 2 | 96.33 | 100 |
|  | 100829 | 17061 - 3 | 88 | 100 |
| CG16992 (mthl6) | 47948 | 16992 - 1 | 94 | 100 |
|  | 47949 | 16992 - 2 | 99.33 | 100 |
| CG3022 (GABA-B-R3) | 50176 | 3022 - 1 | 86 | 100 |
|  | 108036 | 3022 - 2 | 86.33 | 100 |
| CG6706 (GABA-B-R2) | 1784 | 6706 - 1 | 91.66 | 99.66 |
|  | 1785 | 6706 - 2 | 100 | 100 |
| CG15274 (GABA-B-R1) | 15274R-1 II | 15274 - 1 | 100 | 100 |
|  | 15274R-2 III | 15274 - 2 | 96 | 100 |
|  | 101440 | 15274 -3 | 91.66 | 100 |
|  | 105863 | 15274 - 4 | 100 | 100 |
| CG43795 | 17268 | 43795 - 1 | 97.33 | 99 |
|  | 107979 | 43795 - 2 | 72.33 | 100 |
|  | 36156 | 43795 - 3 | 73.33 | 100 |
| CG31760 | 100904 | 31760 - 1 | 85.33 | 100 |
|  | 7686 | 31760 - 2 | 100 | 100 |
| CG30361 (mtt) | 102982 | 30361 - 1 | 82.33 | 100 |
| CG11144 (mGluRA) | 103736 | 11144 - 1 | 96.33 | 100 |
|  | 1793 | 11144 - 2 | 67.66 | 99 |
|  | 1794 | 11144 - 3 | 90 | 99 |
|  | 11144R-3 III | 11144 - 4 | 79.33 | 100 |
|  | 11144R-2 II | 11144 - 5 | 90 | 99 |
| CG32447 | NONE | NA | NA | NA |
| CG31660 (pog) | 7852 | 31660 - 1 | 100 | 100 |
| CG17697 (fz) | 17697R-1 II | 17697 - 1 | 86.66 | 100 |
|  | 17697R-2 X | 17697 - 2 | 84 | 100 |
|  | 43075 | 17697 - 3 | 91 | 100 |
| CG9739 (fz2) | 9739R-1 II | 9739 - 1 | 63.33 | 100 |
|  | 9739R-2 II | 9739 - 2 | 86.66 | 100 |
| CG4626 (fz4) | 5451 | 4626 - 1 | 92 | 96 |
| CG11561 (smo) | 11561R-1 III | 11561 - 1 | 93.33 | 100 |
|  | 11561R-2 X | 11561 - 2 | 89.66 | 100 |
| CG16785 (fz3) | NONE | NA | NA | NA |
| CG1004 (rho) | 1004R-3 III | 1004 - 1 | 93.33 | 100 |
| CG1214 (ru) | 1214R-1 II | 1214 - 1 | 92.33 | 99 |
|  | 1214R-2 III | 1214 - 2 | 100 | 100 |
| CG33166 (stet) | 12083R-1 III | 33166 - 1 | 82.66 | 94 |
|  | 12083R-3 III | 33166 - 2 | 96.66 | 100 |
|  | 7434 | 33166 - 3 | 100 | 100 |
|  | 7435 | 33166 - 4 | 93.33 | 100 |
| CG1697 (rho-4) | NONE | NA | NA | NA |
| CG5634 (dsd) | 5634R-1 X | 5634 - 1 | 100 | 100 |
|  | 5634R-2 X | 5634 - 2 | 85 | 100 |
| CG17212   (rho-6) | NONE | NA | NA | NA |
| CG8972 (rho-7) | 45845 | 8972 - 1 | 98.33 | 100 |
|  | 45846 | 8972 - 2 | 95 | 100 |
| CG8285 (boss) | 8285R-1 on III | 8285 - 1 | 89.33 | 100 |
|  | 8285R-2 on II | 8285 - 2 | 87.66 | 100 |
| CG11895 (stan) | 51379 | 11895 - 1 | 98 | 100 |
|  | 11895R-1 II | 11895 - 2 | 91.66 | 99 |
|  | 11895R-1 X | 11895 - 3 | 100 | 100 |
| CG6919 (oa2) | 6919R-2 II | 6919 - 1 | 70 | 99 |
|  | 6919R-3 II | 6919 - 2 | 80.66 | 100 |
|  | 47895 | 6919 - 3 | 68 | 100 |
|  | 47896 | 6919 - 4 | 91.66 | 100 |
| CG33976 (Octβ2R) | 104050 | 33976 - 1 | 100 | 100 |
|  | 104524 | 33976 - 2 | 95.33 | 99 |
| CG42244 (Octβ3R) | 9068 | 42244 - 1 | 96.33 | 99 |
|  | 101189 | 42244 - 2 | 72 | 100 |
|  | 102515 | 42244 - 3 | 100 | 100 |
| CG7431 (TyrR) | 103572 | 7431 - 1 | 91.66 | 100 |
| CG7994 | 51385 | 7994 - 1 | 91.66 | 100 |
|  | 51427 | 7994 - 2 | 100 | 100 |
| CG16766 (TyrRII) | 16766R-1 III | 16766 - 1 | 82.33 | 100 |
|  | 16766R-3 II | 16766 - 2 | 86.33 | 100 |
|  | 51387 | 16766 - 3 | 98 | 100 |
| CG12796 | 11464 | 12796 - 1 | 97.66 | 100 |
|  | 11465 | 12796 - 2 | 88.66 | 100 |
| CG18314 (DopEcR) | 103494 | 18314 - 1 | 77 | 100 |
| CG10001 (AR-2) | 108648 | 10001 - 2 | 95.33 | 100 |
| CG2872 (AlstR) | 101395 | 2872 - 1 | 80.66 | 100 |
|  | 48496 | 2872 - 2 | 100 | 100 |
|  | 39221 | 2872 - 3 | 99 | 100 |
|  | 3399 | 2872 - 4 | 100 | 100 |
| CG34411 | 4187R-1 II | 34411 - 1 | 96.33 | 100 |
|  | 4187R-4 II | 34411 - 2 | 79.33 | 100 |
|  | 102681 | 34411 - 3 | 89.66 | 99 |
|  | 108915 | 34411 - 4 | 90 | 100 |
| CG31096 (Lgr3) | NONE | NA | NA | NA |
| CG7665 (Lgr1) | 7665R-1 II | 7665 - 1 | 81.66 | 100 |
|  | 7665R-3 II | 7665 - 2 | 48.33 | 50 |
|  | 107823 | 7665 - 3 | 98.33 | 100 |
| CG8930 (rk) | 8930R-1 II | 8930 - 1 | 100 | 100 |
|  | 8930R-2 II | 8930 - 2 | 95 | 99 |
| CG42301 (CCKLR-17D1) | 6857R-1 II | 6857 - 1 | 96.66 | 100 |
|  | 100760 | 6857 - 2 | 100 | 100 |
| CG32540 CCKLR-17D3 | 6881R-1 (II) | 6881 - 1 | 95.33 | 100 |
|  | 110484 | 6881 - 2 | 95 | 100 |
| CG8784 | 15989 | 8784 - 1 | 96.66 | 100 |
|  | 103822 | 8784 - 2 | 100 | 100 |
| CG8795 | 8795R-1 III | 8795 - 1 | 98.33 | 99.66 |
|  | 1768 | 8795 - 2 | 100 | 100 |
|  | 44871 | 8795 - 3 | 81.66 | 96.33 |
|  | 100927 | 8795 - 4 | 84 | 100 |
| CG9918 (Pk1r) | 101115 | 9918 - 1 | 87.66 | 100 |
| CG10698 (CrzR) | 44310 | 10698 - 1 | 92.66 | 100 |
|  | 108506 | 10698 - 2 | 99.33 | 100 |
| CG1147 (NPFR1) | 9605 | 1147 - 1 | 76 | 100 |
|  | 107663 | 1147 - 2 | 84 | 93.33 |
|  | 1147R-1 III | 1147 - 3 | 88.33 | 100 |
|  | 1147R-3 II | 1147 - 4 | 100 | 100 |
| CG5911 (ETHR) | 42716 | 5911 - 1 | 100 | 100 |
|  | 101996 | 5911 - 2 | 91.33 | 100 |
| CG10823 (SIFR) | 1783 | 10823 - 1 | Pupal lethal | 100 |
|  | 25831 (BDSC) ⬩ | 10823-2 | 98 | 100 |
| CG13575 | 9362 | 13575 - 1 | 99 | 100 |
|  | 9363 | 13575 - 2 | 81.66 | 100 |
|  | 103435 | 13575 - 3 | 91 | 100 |
| CG34381 (TrissinR) | 7886 | 34381 - 1 | 88.33 | 100 |
|  | 107943 | 34381 - 2 | 100 | 100 |
|  | 42758 | 34381 - 3 | 96.66 | 100 |
| CG10626 (Lkr) | 22845 | 10626 - 1 | 100 | 100 |
|  | 105155 | 10626 - 2 | 94.33 | 100 |
| CG6515 (Takr86C) | 13392 | 6515 - 1 | 60 | 100 |
|  | 107090 | 6515 - 2 | 100 | 100 |
| CG5811 (NepYr) | 1258 | 5811 - 1 | 86 | 100 |
|  | 1259 | 5811 - 2 | 96.66 | 100 |
|  | 103973 | 5811 - 3 | 95.66 | 100 |
| CG7887 (Takr99D) | 1372 | 7887 - 1 | 100 | 100 |
|  | 44369 | 7887 - 2 | 100 | 100 |
| CG3856 (Oamb) | 3856R-1 III | 3856 - 1 | 90 | 100 |
|  | 2861 | 3856 - 2 | 93.33 | 100 |
|  | 106511 | 3856 - 3 | 100 | 100 |
| CG8985 (DmsR-1) | 9370 | 8985 - 1 | 78.33 | 100 |
|  | 8985R-2 II | 8985 - 2 | 71.33 | 100 |
| CG14593 (CCHa2r) | 1658 | 14593 - 1 | 95.66 | 100 |
|  | 107481 | 14593 - 2 | 100 | 100 |
| CG30340 | 7387 | 30340 - 1 | 100 | 100 |
|  | 100088 | 30340 - 2 | 100 | 100 |
| CG32447 | 5417 | 32447 - 1 | 97.66 | 100 |
|  | 102740 | 32447 - 2 | 95 | 100 |
| CG30106 (CCHa1r) | 1678 | 30106 - 1 | 95 | 100 |
|  | 103055 | 30106 – 2 | 72.33 | 100 |
| Elav^C155^GAL4 | NA | Elav^C155^GAL4 | NA | 100 |

⌃NIG/VDRC: National Institute of Genetics Fly Stocks Centre, Kyoto, Japan/ Vienna *Drosophila* RNAi center, Vienna, Austria

⬩BDSC: Bloomington *Drosophila* Stock center, Bloomington, USA
